# Dietary Modifications in IBS Leads to Reduced Symptoms, Weight, and Lipid Levels: Two Randomized Clinical Trials

**DOI:** 10.3390/nu17182966

**Published:** 2025-09-16

**Authors:** Bodil Roth, Bodil Ohlsson

**Affiliations:** 1 Department of Clinical Sciences, Lund University, 22100 Lund, Sweden; bodil.roth@med.lu.se; 2 Department of Internal Medicine, Skåne University Hospital, 20502 Malmö, Sweden

**Keywords:** dietary habits, irritable bowel syndrome, lipid profile, low FODMAP, SSRD, symptoms, weight

## Abstract

**Background/Objectives**: Irritable bowel syndrome (IBS) is presented with both gastrointestinal and extraintestinal symptoms. In addition, overweight/obesity and metabolic syndrome is prevalent in IBS. Dietary interventions with a low content of fermentable oligo-, di-, and monosaccharides and polyols (FODMAP) or a starch- and sucrose-reduced diet (SSRD) efficiently reduce symptoms and weight. Our hypothesis was that not only nutrition composition but also weight reduction is of importance for symptom relief. The aim was to merge two randomized trials and examine symptoms, weight, nutrition intake, and lipid levels at baseline and during nutritional intervention. **Methods**: One study with 105 IBS patients randomized to either an SSRD (*n* = 80) or control diet (*n* = 25) and one study with 155 IBS patients randomized to an SSRD (*n* = 77) or low FODMAP (*n* = 78) were merged. Symptom and food questionnaires were analyzed together with weight/body mass index (BMI) and lipid levels. **Results**: Patients had moderate or severe IBS at baseline, and half of them were overweight/obese. Energy intake was reduced by both diets, with the most pronounced carbohydrate reduction after the SSRD. The cholesterol levels were highest in the second cohort, possibly due to the higher fat and lower starch intake. About 25% had high-density lipoprotein below reference levels. Gastrointestinal and extraintestinal symptoms, as well as weight/BMI, were reduced by the SSRD and low FODMAP, but not in the control group. The SSRD in the second cohort and low FODMAP rendered lower levels of total cholesterol, low-density lipoprotein, and non-high-density lipoprotein levels. Weight/BMI were more often associated with lipid levels and symptoms than nutrient composition at baseline, and weight/BMI reductions correlated with carbohydrate reduction and were associated with a reduction in gastrointestinal and extraintestinal symptoms. **Conclusions**: Not only food components, but also overweight/obesity may be of importance for the development and severity of IBS and related symptoms.

## 1. Introduction

Disorders of gut–brain interaction (DGBI), defined according to the Rome IV criteria [1], are common with a 40% prevalence of functional gastrointestinal (GI) symptoms in the general population [2]. Irritable bowel syndrome (IBS) is most common, with a global prevalence of 4% [1,2]. The etiology of DGBI is unknown, but risk factors such as female sex, trauma, abuse, lifetime stress, psychological disorders, and a family history of IBS have been identified [3]. An association of IBS with overweight, obesity, and metabolic syndrome has been noticed [3,4,5,6]. The most efficient way to treat IBS is dietary treatments, according to the National Institute for Health and Care Excellence (NICE) guidelines, and low content of fermentable oligo-, di-, and monosaccharides and polyols (FODMAP) [7,8]. Recently, a low-carbohydrate diet has been proven to be as efficient as low FODMAP [9]. However, the low-FODMAP diet is difficult to adhere to [8], and a very-low-carbohydrate diet may lead to hyperlipidemia [9].

Congenital sucrase-isomaltase deficiency (CSID) is a genetic disease leading to GI symptoms in children [10]. CSID is rare, except in Arctic populations, with a prevalence of almost 10% [11]. An increased prevalence of rare variants of sucrase-isomaltase (*SI*) genes has been found in IBS patients [12,13], in alignment with an improvement in GI symptoms using a starch- and sucrose-reduced diet (SSRD) [14,15]. Surprisingly, different diets like low FODMAP, SSRD, and low-carbohydrate diets render similar responder rates [9,16].

In population-based studies, Greenlandic adults with *SI* variants were found to have lower weight, body mass index (BMI), fat percentage, and liver enzymes, and a healthier lipid profile, compared with other individuals [11]. A hypothesis-confirming animal study found that *SI* knockout mice were leaner and had lower triglyceride levels on a sucrose-containing diet compared with wild-type mice [11]. We found similar results of weight reduction with improved symptoms and nutrient profile after a 4-week dietary intervention with SSRD [14,16,17].

Our hypothesis was that not only is food composition of importance for symptom improvements in IBS during a dietary intervention, but also weight reduction may be of importance for symptom reduction and improvement of the metabolic profile. To obtain greater power, two randomized IBS trials were merged [14,16]. The primary aim of the present study was to examine symptoms, weight, BMI, and lipid levels before and after a dietary intervention and evaluate the associations between nutrient intake and body composition with symptoms and lipid levels at baseline and during the intervention. The secondary aim was to compare the effects between SSRD, low FODMAP, and controls regarding nutrient intake, symptoms, weight/BMI, and lipids.

## 2. Materials and Methods

### 2.1. Study Design

**Figure 1:**
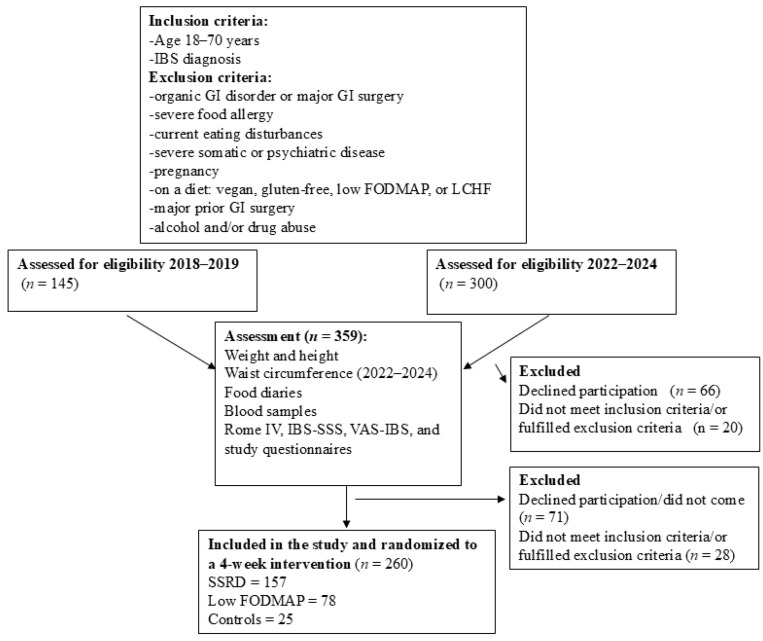
Flow chart of the recruitment process. GI = gastrointestinal. IBS-SSS = irritable bowel syndrome severity scoring system. LCHF = low-carbohydrate high-fat diet. Low FODMAP = low content of fermentable oligo-, di-, and monosaccharides and polyols. n = number. SSRD = starch- and sucrose-reduced diet. VAS-IBS = visual analog scale for irritable bowel syndrome. 2018–2019 means the first cohort [14], and 2022–2024 means the second cohort [16].

Two open randomized trials, with two parallel groups, were performed at the Department of Internal Medicine, Skåne University Hospital, Malmö, Sweden, during 2018–2019 and 2022–2024, respectively. A dietary intervention was given for 4 weeks, where the SSRD was compared with ordinary food without any dietary changes in the first trial in 2018–2019 and with low FODMAP in the second trial in 2022–2024. Study questionnaires, diary books, and blood samples were collected at baseline and after study completion. Weight was measured at both visits and BMI calculated and defined according to the World Health Organization (WHO) [18]. Waist circumference was measured in the second cohort and visceral adiposity defined according to guidelines for metabolic syndrome [19] (Figure 1).

### 2.2. Patients

The inclusion criteria were an age of 18 to 70 years and a diagnosis of IBS. The presence of insufficient symptoms, i.e., scores <175 on the irritable bowel syndrome severity scoring system (IBS-SSS) [20], alcohol or drug abuse, current eating disturbances, pregnancy, the presence of any organic GI disease, severe GI surgery in the past, severe food allergy, severe organic and psychiatric diseases, or on gluten-free, vegan, low-FODMAP, or low-carbohydrate high-fat (LCHF) diets were identified as exclusion criteria (Figure 1).

The first cohort was included between January 2018 and February 2019 and was described in detail previously [14]. Briefly, patient registries were provided by Region Skåne, with all subjects who had been given an IBS diagnosis code of K58.0 (diarrhea-predominated IBS: IBS-D) or K58.9 (IBS) according to the International Statistical Classification of Diseases and Related Health Problems—ICD-10 in primary health care centers (PCCs) during 2015–2017 or the Department of Gastroenterology and Hepatology during 2016–2017. In total, 1039 unique IBS patients from PCCs were identified. Invitation letters were sent to 528 randomly selected patients. From the tertiary care center, 640 unique patients were identified. Out of these, 151 patients were randomly selected to receive invitation letters. Patients were called a few weeks later (BR). After the provision of further verbal information, 145 patients first agreed to participate (112 patients (77%) from PCCs; 34 males (23%)). After this initial acceptance, 40 patients (29 women (72%)) were later excluded because they did not show up or were no longer willing to participate (*n* = 18), had mild symptoms (*n* = 14), wrong diagnoses (*n* = 5), or were already on a diet meeting the exclusion criteria (*n* = 3). Thus, 105 patients (82 women (78%)) were eventually included in the study (77 patients (73%) from PCCs) from the 679 invitation letters sent (15% inclusion rate) (Supplementary Figure S1).

The second study was conducted between March 2022 and February 2024 and was described in detail previously [21]. Briefly, a data search was performed at Clinical Studies Sweden—Regional node for Southern Sweden from medical records in the County of Skåne using the revised ICD-10 to identify patients who had received any of the diagnoses K58.1 (IBS-D), K58.2 (constipation-predominated IBS: IBS-C), K58.3 (mixed IBS: IBS-M), and K58.8 (other or unspecified IBS: IBS-U) during 2019–2022 [22]. Of the 3587 patients who lived close to Malmö, 744 were randomly selected to be contacted by a written letter to inform them about the study. They were asked to provide contact details to the investigators and encouraged to call or email the investigators if they were interested in participating (BR). Out of these, 58 were willing to participate.

Information letters with leaflets were sent to 203 PCCs in the county for distribution to patients, and several lectures were given in person or digitally for healthcare staff (BR and BO). Three referrals were obtained from the healthcare system. Eight patients contacted the investigators after being encouraged by their general practitioner (GP) or the dietician at the PCC to participate. Information leaflets left in the waiting rooms of the PCCs led to nine patients contacting the investigators, and four patients contacted the investigators after recommendations from friends or relatives. Of these 24 patients, 8 were excluded due to the exclusion criteria. Two campaigns were performed on social media by a professional company (Trialy, Gothenburg, Sweden). The 218 patients who had been assigned to participate in the study were contacted by phone by one of the investigators (BR), which led to 140 patients who did not meet any exclusion criteria and were willing to participate.

Altogether, 214 patients were randomized to either low FODMAP or SSRD according to block randomization (BR). Out of these, some did not attend the first-time appointment (*n* = 53), did not fulfill the inclusion criteria (*n* = 3), or met the exclusion criteria (*n* = 3) (Figure 1). Seven of the included IBS patients had total IBS-SSS just below 175 but were still included due to typical symptoms and clear diagnosis. Finally, 155 IBS patients (72.4% of randomized cases) were admitted to the dietary intervention (BO) (Supplementary Figure S2). This renders an inclusion rate of 42.7% in the group recruited from social media and 6.5% in the group recruited from medical records.

Combining the two cohorts rendered 260 patients: 157 randomized to SSRD, 78 randomized to low FODMAP, and 25 randomized to controls without any dietary restrictions (Figure 1).

### 2.3. Dietary Advice

Patients received verbal and written dietary information about the diets. Patients randomized to the SSRD had to focus on the reduction of starch and sucrose, and increased intake of meat, fish, dairy products, and fruits and vegetables low in starch (Supplementary Tables S1 and S2) (BO). The dietary advice was modified from guidelines for patients with CSID [23] and previously described in more detail [14]. Briefly, sucrose-containing foods should be avoided. One serving per day was allowed for whole-grain bread or oatmeal porridge. Whole grains from oats, barley, and bran were recommended instead of processed breakfast cereals. Fiber-rich pasta and rice should be chosen. Small amounts of processed rice and pasta once daily could be consumed for participants not tolerating fibers. Pork, beef, lamb, fish, turkey, chicken, and egg could be ingested without any restrictions. Processed meat treated with sugar or starch such as bacon, pies, and sausage should be avoided. Natural dairy products could be ingested without restrictions, whereas oat milk and soy milk were not allowed. Butter and oil intake was unrestricted. Milk, sugar-free soda, and home-made juices were allowed if the juice was made from recommended fruits. Salt, pepper, and fresh herbs could be used unrestrictedly. Nuts and seeds were recommended in place of sugary snacks. Increased fat and/or protein intake and prolonged chewing were encouraged to enhance the salivary amylase breakdown of starch and to delay GI transport. No advice was provided regarding meal frequency or meal regularity.

Participants randomized to the low FODMAP received written and oral information about the diet. They were informed to avoid or reduce their intake of fructans (e.g., wheat, onion, garlic), galacto-oligosaccharides (e.g., pulses (pea, lentil, faba bean, and lupin)), lactose (e.g., milk), fructose more than glucose (e.g., honey), and polyols (e.g., apples, pears) during the 4-week intervention [24]. After the 4 weeks, they had to reintroduce FODMAP-containing food again, to finally identify their personalized form of diet according to clinical routines [25,26].

Recipes and suggestions on the menu to enable compliance with the diet were provided to all participants (BR). All participants were encouraged to continue with their ordinary energy intake, degree of physical activity, medications, and probiotics or supplements, without making any changes or to introduction of any new drugs or other dietary changes. The participants could call or email the study staff whenever they wanted during the study.

### 2.4. Questionnaires

#### 2.4.1. Study Questionnaire

All study participants were asked to complete a questionnaire regarding sociodemographic factors, lifestyle habits, pregnancies and childbirth, medical history, drug treatments, family history, and previous and current dietary modifications. In the first cohort, participants reported amount and/or volume of all consumed foods for 4 consecutive days (Wednesday–Saturday) at baseline and at the end of the intervention. They had to describe the percentage of fat in dairy products, fiber in bread products, cacao in chocolate, and information on sugar-free or regular soda consumed. Nutrient intake in amount and energy percentages (E%) was calculated from a single day (day 2) of the 4-day registrations by a nutritionist, using the AIVO Diet computer program, Stockholm, Sweden [27]. In the second cohort, a food diary book was completed digitally for 3 days (Wednesday–Friday) at baseline and at week 4 on a platform called Riksmaten Flex 2021 of the Swedish Food Agency [28]. Nutrient intake was calculated for the mean daily intake during the 3 days.

#### 2.4.2. Rome IV Questionnaire

Question numbers 40–48 in the Swedish version of the ROME IV questionnaire were used to diagnose IBS [29] after having received a license from The Rome Foundation, Inc., Raleigh, NC, USA.

#### 2.4.3. Irritable Bowel Syndrome Severity Scoring System

IBS-SSS assesses abdominal pain, abdominal distension, satisfaction with bowel habits, and the impact of bowel habits on daily life using a visual analog scale (VAS) ranging from absent (0 mm) to very severe (100 mm) symptoms. The number of days with abdominal pain in the last 10 days was reported. The maximum achievable score is 500. Scores ranging from 75 to 174 indicate mild disease, 175–299 indicate moderate disease, and ≥300 indicate severe disease. Extraintestinal symptoms (nausea, difficulties eating a whole meal, headache, back pain, fatigue, belching/excess wind, reflux, urinary urgency, leg pain, and muscle/joint pain) were assessed on VASs with a maximal achievable score of 500 [20].

#### 2.4.4. Visual Analog Scale for Irritable Bowel Syndrome

VAS-IBS assesses the influence of abdominal pain, diarrhea, constipation, bloating and flatulence, vomiting and nausea, psychological well-being, and intestinal symptoms on daily life, ranging from absent (0 mm) to very severe (100 mm) symptoms. The values are inverted from the original format and validated to measure changes over time [30,31].

### 2.5. Laboratory Analyses

Cholesterol, high-density lipoprotein (HDL) cholesterol, low-density lipoprotein (LDL) cholesterol, and non-HDL cholesterol were analyzed in plasma according to clinical routines at the Department of Clinical Chemistry [32]. HDL levels were defined as below or above the levels given for the definition of metabolic syndrome [19].

### 2.6. Statistical Analysis

The primary outcome for each study was the responder rate (RR) of improved GI symptoms, defined as a decrease in total IBS-SSS to at least 50 points (RR = ∆Total IBS-SSS ≥ −50). The first intervention was conducted as a pilot trial. In the second IBS intervention, power calculation was based on non-inferiority where the new SSRD diet was tested against the standard treatment of low FODMAP. Primary outcome was responder rate (RR = ∆Total IBS-SSS ≥ −50) and was assumed to be 65% in both treatment groups. A difference in responder rate as large as 20% in favour of low FODMAP would allow the new SSRD treatment to be non-inferior. Sample size based on 80% power, a one-sided confidence level of 97.5%, and an expected loss of follow up of 10% to confirm non-inferiority was calculated to be 100 patients in each group. Due to a few dropouts at follow up, the study was completed after the inclusion of 155 patients after a second consultation with the statistician.

The statistical calculations were performed in IBM SPSS, version 29, IBM, New York, NY, USA. Due to no or small differences between the two cohorts regarding sociodemographic factors and lifestyle habits, the two groups were calculated together. Since variable data were not normally distributed according to the Kolmogorov–Smirnov test, data were presented as median (interquartile ranges) or number (percentages). Mann–Whitney U and Kruskal–Wallis tests were used for comparisons between groups of baseline values and differences from 4 weeks to baseline, and Wilcoxon Signed Ranks for comparisons within groups. Spearman’s correlation test was used for correlations and Fisher’s exact test was used for dichotomous variables. To adjust for multiple comparisons in the correlations, crude *p*-values as well as the *q*-values adjusted for false discovery rate (FDR) set at 5% according to the Benjamini–Hochberg method were calculated within each hypothesis [33]. The FDR-adjusted *q*-values were the main results. Associations between symptoms and lipids (dependent variables) with weight and nutrient intake (independent variables) were calculated with generalized linear models, using a first model with weight, carbohydrates, protein, fat, and fiber, and a second model with weight, starch, and sucrose. Values were given as β value and 95% confidence interval (CI). Calculations were performed per protocol and missing values were excluded from analyses. *p* < 0.05 or *q* < 0.05, when applicable, was considered statistically significant.

## 3. Results

### 3.1. Basal Characteristics

Altogether, 260 patients were included in the study. Of these, 46 (17.7%) patients had IBS-C, 70 (26.9%) patients had IBS-D, 91 (35.0%) patients had IBS-M, 10 (3.8%) patients had IBS-U, and 41 (14.8%) patients had unspecific functional bowel disorder (FBD) with weekly abdominal pain but a weak association (<30%) between the pain and bowel habits. Two participants in the first cohort did not complete the Rome IV questionnaire.

Regarding improvements in GI symptoms, the RR in the first cohort was 57 subjects (71.3%; 95% CI: 0.663–0.851) in the SSRD group and six subjects (24.0%; 95% CI: 0.120–0.449) in the control group (*p* < 0.001). In the second cohort, the RR was 61 subjects (84.7%; 95% CI: 0.747–9.912) in both diet groups (*p* = 1.000).

Both weight and BMI were equal between the two cohorts (71 (63–82) kg and 69 (63–83) kg; *p* = 0.718 and 24 (22–28) kg/m^2^ and 25 (23–28) kg/m^2^; *p* = 0.808, respectively). There was no difference in physical activity between the two cohorts (*p* = 0.636). Hypothyroid disease was more common in the first cohort (11.4% vs. 3.9%, *p* = 0.024), whereas eczema was more common in the second cohort (4.8% vs. 12.3%, *p* = 0.049). Levaxine treatment was more commonly used in the first cohort, and laxatives and proton pump inhibitors were more common in the second cohort (Supplementary Table S3).

Next, 157 patients were randomized to the SSRD (10 lost at follow up), 78 to low FODMAP (6 lost at follow up), and 25 as controls (2 lost at follow up) who had to continue with their ordinary food habits during the intervention (Supplementary Figures S1 and S2). The only differences between the three groups were that more in the control group were living alone and were more often smokers (Table 1).

### 3.2. Food Intake

At baseline, the intake of starch was higher in the first IBS cohort (76 (48–111) g and 45 (31–63) g, *p* < 0.001, respectively), with higher fat intake in the second cohort (62 (45–91) g and 75 (57–93) g, respectively, *p* = 0.004) (Supplementary Table S4). The starch intake was reduced in both cohorts during the intervention (*p* < 0.001 for both). The total fat intake was significantly increased in the first cohort during the intervention (*p* = 0.049), but not in the second cohort (*p* = 0.141). The increase was due to an increased intake of polyunsaturated fat, which was statistically significant in both cohorts (*p* = 0.017 and *p* = 0.013, respectively).

Energy intake was reduced after 4 weeks in both the SSRD group and the low FODMAP group. During the intervention, the intake of carbohydrate, sucrose, and starch was markedly reduced in the SSRD group, with a smaller reduction also observed after low FODMAP regarding carbohydrates and sucrose, which differed between groups (*p* < 0.001 for all). The fiber intake was reduced in both groups. Both protein and fat intakes were increased in the SSRD group, but were unaffected in the low FODMAP group. The fat increase in the SSRD group was due to an increased intake of mono-unsaturated and poly-unsaturated fatty acids compared to the other groups. No dietary changes were observed in the control group (Table 2).

The proportion of patients with a reduced carbohydrate intake of ≥30% was 105 subjects (66.9%) in the SSRD group, 17 (21.8%) in the low FODMAP group, and 7 (28.0%) in the control group (*p* < 0.001). The corresponding proportion for starch was 110 subjects (70.1%) in the SSRD group, 20 (25.6%) in the low FODMAP group, and 9 (36.0%) in controls (*p* < 0.001). Regarding sucrose intake, the proportions were 114 subjects (72.6%) in the SSRD group, 28 (35.9%) in the low FODMAP group, and 11 (44.0%) in the controls (*p* < 0.001).

### 3.3. Weight and BMI

**Figure 2:**
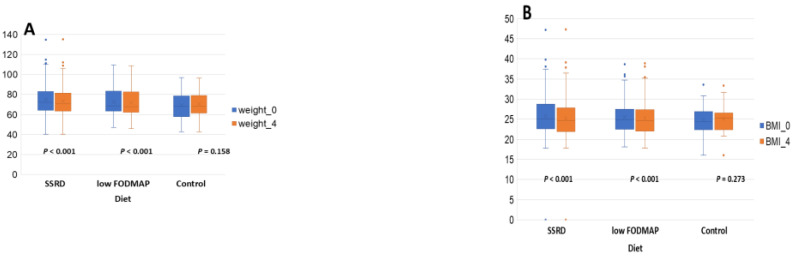
The changes in weight (**A**) and body mass index (BMI) (**B**) before and after a 4-week intervention. SSRD = starch-and sucrose-reduced diet. Low FODMAP = low content of fermentable oligo-, di-, and monosaccharides and polyols. There were 17 missing in the SSRD group, 6 missing in the low FODMAP group, and 4 missing in the control group. Dots represent outliers. Wilcoxon Signed Rank test. *p* < 0.05 was considered statistically significant.

Due to the reduced energy intake during the active interventions (Table 2), weight and BMI were reduced during the study, which was not found in the control group (Figure 2, Supplementary Table S5). At baseline, 127 patients (48.8% (50.4% per protocol)) were normal weight, 90 (34.6% (35.7% per protocol)) were overweight, and 35 (13.5% (13.9% per protocol)) were obese. After 4 weeks, 127 (48.8% (54.0% per protocol)) were normal weight, 79 (30.3% (33.8% per protocol)) were overweight, and 29 (11.5% (12.4% per protocol)) were obese, which did not differ between diets (*p* = 0.957) or between baseline and 4 weeks (*p* = 0.726). Waist circumference was only measured in the latter cohort, where 79 (60.8%) of women and 17 (68.0%) of men had visceral obesity at baseline, with a reduction after 4 weeks with 74 (56.9% (61.2% per protocol)) women and 11 men (44.0% (50% per protocol)), which did not differ between diets (*p* = 0.735) or time points (*p* = 0.635). There were correlations between weight and energy intake (Rs = 0.171, *q* = 0.028), carbohydrate intake (Rs = 0.148, *q* = 0.042), and fat intake (Rs = 0.167, *q* = 0.028) at baseline, and reduced weight and BMI during the study correlated with reduced intake of energy (*q* = 0.026 vs. q = 0.014), carbohydrates (*q* = 0.004 for both), starch (*q* = 0.004 for both), and sucrose (*q* = 0.035 vs. *q* = 0.028) (Table 3).

### 3.4. Gastrointestinal and Extraintestinal Symptoms

At baseline, the degree of symptoms was equal between the groups. All GI and extraintestinal symptoms were markedly reduced in both intervention groups (*p* < 0.001 for all), whereas only constipation and bloating and flatulence decreased in the control group (Table 4 and Table 5). After 4 weeks, the reductions in abdominal pain, bloating and flatulence, intestinal symptoms’ influence on daily life, psychological well-being, and total IBS-SSS differed between the groups (Table 4). Regarding extraintestinal symptoms, the reductions in fatigue, belching/excess wind, muscle/joint pain, and total extraintestinal IBS-SSS differed between groups (Table 5).

The only correlation of GI symptoms at baseline that remained after FDR adjustment was an inverse correlation between weight and constipation (*q* = 0.008) (Supplementary Table S6). Reduction in carbohydrate intake corelated with reduced bloating and flatulence (*q* = 0.032) and total IBS-SSS (*q* = 0.032) (Supplementary Table S7). Regarding extraintestinal symptoms at baseline, weight correlated with back pain and muscle/joint pain (both *q* = 0.035), whereas BMI correlated with back pain (*q* = 0.003), fatigue (*q* = 0.018), leg pain (*q* = 0.018), muscle/joint pain (*q* = 0.003), and total amount of total extraintestinal IBS-SSS (*q* = 0.003) (Supplementary Table S8). Reduced energy intake correlated with reduced back pain (*q* = 0.010), reduced belching/excess wind (*q* = 0.030), and total extraintestinal IBS-SSS (*q* = 0.047), and reduced fat intake correlated with reduced back pain (*q* = 0.030) (Supplementary Table S9).

### 3.5. Laboratory Analyses

The cholesterol values differed between the two IBS cohorts and were therefore calculated separately, with higher values of cholesterol (4.60 (4.00–5.28) mmol/L and 4.90 (4.40–5.70) mmol/L, respectively, *p* = 0.013), HDL cholesterol (1.40 (1.20–1.60) mmol/L and 1.60 (1.20–1.80) mmol/L, respectively, *p* < 0.001), and LDL cholesterol (2.40 (1.82–2.88) mmol/L and 3.20 (2.70–3.80) mmol/L, respectively, *p* < 0.001) in the second cohort (Supplementary Table S4). Cholesterol, LDL cholesterol, and non-HDL cholesterol values were reduced after SSRD and low FODMAP in the second IBS cohort, but SSRD did not reduce lipid levels in the first cohort with initially lower baseline values (Table 6). In the first cohort, 28 subjects (26.7% (26.9% per protocol)) had HDL levels below reference values for metabolic syndrome, whereas in the latter cohort, 34 subjects (21.9% (22.4% per protocol)) had lowered HDL levels [19]. After 4 weeks, the corresponding figures were 19 (18.1% (20.7% per protocol)) and 29 (18.7% (20.4% per protocol)), which did not differ between diets (*p* = 0.912) or time points (*p* = 0.332). Weight and BMI correlated positively with LDL (*q* = 0.020 vs. *q* = 0.001) and non-HDL cholesterol (*q* = 0.002 vs. *q* = 0.001) and inversely with HDL cholesterol (*q* = 0.002 vs. *q* = 0.001) at baseline. BMI also correlated with total cholesterol levels (*q* = 0.025). Starch correlated inversely with both LDL (*q* = 0.004) and HDL cholesterol (*q* = 0.006). During the intervention, the reduced weight and BMI correlated with reduced HDL cholesterol (*q* = 0.024 vs. *q* = 0.028) (Supplementary Table S10).

### 3.6. Associations Between Nutrients, Weight, Symptoms, and Lipid Levels

#### 3.6.1. First Model

Calculation with the model of weight, carbohydrates, protein, fat, and fiber at baseline showed that weight was associated with LDL cholesterol and non-HDL cholesterol but inversely associated with HDL cholesterol. Further, weight was associated with several extraintestinal symptoms but negatively associated with the intestinal symptoms’ influence on daily life. There were positive associations between fat intake and cholesterol and LDL cholesterol levels, whereas carbohydrate and protein intakes were inversely associated with LDL cholesterol. Only a few weak associations were observed between nutrient intake and extraintestinal symptoms (Supplementary Table S11).

Weight reduction was associated with reduced HDL cholesterol (β: 0.017; 95% CI: 0.006–0.029); *p* = 0.004), intestinal symptoms’ influence on daily life (β: 2.022; 95% CI: 0.015–4.028, *p* = 0.048), total IBS-SSS (β: 7.241; 95% CI: 0.071–14.412; *p* = 0.048), total extraintestinal IBS-SSS (β: 5.169; 95%: 0.807–9.530; *p* = 0.020), and the specific symptoms of fatigue, reflux, and urinary urgency. The reduced intake of carbohydrates was associated with the reduced total IBS-SSS (β: 0.218; 95% CI: 0.017–0.420; *p* = 0.034) and headache (β: 0.062; 95% CI: 0.018–0.106, *p* = 0.006). The changes in protein, fat, and fiber intake were associated with some changes in specific GI and extraintestinal symptoms, but no association was observed between the changes in nutrient intake and total extraintestinal IBS-SSS or lipid levels (Supplementary Table S12).

Similar findings were observed when calculating with BMI instead of weight, except for an association between BMI and total extraintestinal IBS-SSS at baseline (Supplementary Tables S13 and S14).

#### 3.6.2. Second Model

Calculations with the second model, including weight, sucrose, and starch at baseline, showed an inverse association between HDL cholesterol and weight (β: −0.010; 95% CI: −0.013–(−0.007); *p* < 0.001) and starch (β: −0.001; 95% CI: −0.003–0.000; *p* = 0.013), as well as an inverse association between LDL cholesterol and starch (β: −0.006; 95% CI: −0.010–(−0.003); *p* < 0.001) and a positive association between LDL cholesterol and weight (β: 0.011; 95% CI: 0.003–0.020; *p* = 0.007).

Weight was inversely associated with constipation (β: −0.557; 95% CI: −0.824-(−0.290); *p* < 0.001), bloating (β: −0.198; 95% CI: −0.384–(−0.12); *p* = 0.037), and intestinal symptoms’ influence on daily life (β: −0.180; 95% CI: −0.359-4.496E-5); *p* = 0.050), but positively associated with back pain (β: 0.333; 95% CI: 0.068–0.599); *p* = 0.014), fatigue (β: 0.246; 95% CI: 0.018–0.475; *p* = 0.035), urinary urgency (β: 0.281; 95% CI: 0.015–0.548; *p* = 0.039), leg pain (β: 0.296; 95% CI; 0.094–0.479; *p* = 0.004, and muscle/joint pain (β: 0.363; 95% CI: 0.087–0.638; *p* = 0.010). The only association with sucrose intake was an inverse association with urinary urgency (β: −0.239; 95% CI: −0.427–(−0.052); *p* = 0.012).

During the study, weight reduction was associated with decreased HDL cholesterol levels (β: 0.016; 95% CI: 0.023–0.033; *p* = 0.006), total IBS-SSS (β: 7.465; 95% CI: 0.147–14.784; *p* = 0.046), total extraintestinal IBS-SSS (β: 5.243; 95% CI: 0.855–9.632; *p* = 0.019), fatigue (β: 2.282; 95% CI: 0.535–4.030; *p* = 0.010), reflux (β: 1.801; 95% CI: 0.047–3.555; *p* = 0.044), and urinary urgency (β: 2.232; 95% CI: 0.449–4.015; *p* = 0.014). The reduced sucrose intake was associated with reduced total IBS-SSS (β: 0.582; 95% CI: 0.019–1.145; *p* = 0.043).

Calculations with BMI instead of weight showed similar results (Supplementary Tables S15 and S16).

## 4. Discussion

The main findings in the study were that overweight/obesity, visceral adiposity, and low HDL cholesterol levels were found in high prevalence in IBS. GI and extraintestinal symptoms, weight, BMI, and cholesterol levels were reduced after SSRD and low FODMAP. Intake of energy, carbohydrates, and fat correlated with weight at baseline, and reduction in energy, carbohydrates, starch, and sucrose during the study correlated with reduced weight and BMI. Association calculations with models consisting of groups of nutrients together with weight or BMI showed more significant changes than simple correlations. Weight and BMI were more often associated with lipid levels and symptoms than nutrients at baseline, especially extraintestinal symptoms. The lipid levels were weakly associated with carbohydrate, fat, protein, and starch intake. Reduction in weight and BMI were associated with the reduction in total GI and extraintestinal symptoms as well as specific extraintestinal symptoms. The reduced intake of carbohydrates was associated with reduced GI symptoms, where sucrose was of more importance than starch. The exploratory path diagram shown in Supplementary Figure S3 summarizes the hypothesis behind the improvements.

Obesity has been described as a risk factor for abdominal pain related to DGBI in a systematic review [3]. Visceral adiposity is a hallmark of metabolic syndrome [19]. In the present cohort, more than half of the cohort fulfilled the criteria for visceral adiposity, which is in alignment with the association between IBS and central obesity according to Mendelian randomization [6]. In one study, 30% of obese patients also suffered from IBS [34]. However, different prevalences of DGBI in different obesity groups may be explained by different recruitment sources [35]. In addition, the lower HDL levels in one-quarter of patients points to a disturbed metabolic profile. These aspects must be considered in the dietary regimes of DGBI. We must consider that not only is the reduction in symptoms of importance for this patient group, but also the general health aspects with reduction in weight, inflammation, lipid levels, and improved hormone profile as well as other signs of metabolic syndrome, together with a healthier diet with increased intake of micronutrients [5,16,17,36]. Taken together, these findings indicate that an SSRD should be offered to overweight/obese IBS patients as the first choice instead of low FODMAP.

Weight and BMI were associated with more symptoms at baseline, and the weight and BMI reduction was associated with reduced symptoms, whereas the nutrient composition showed fewer associations with symptoms. One can speculate over the association between weight and symptoms. One explanation may be that a lower weight represents a healthier lifestyle with more healthy food and physical activity [37]. IBS has been associated with low-grade inflammation [38]. Since adipose tissue is an endocrine organ that produces several adipokines and proinflammatory cytokines [39,40], weight reduction may lead to a less inflammatory milieu with less risk for the development of metabolic diseases [41]. This has been demonstrated by lower circulating leptin and PAI-1 levels after an SSRD [42]. Similar responder rates in different dietary regimes with healthy eating patterns may be explained by reduced fat mass and a reduced inflammatory state [9,16,17,37].

Historically, most interest has focused on macronutrient composition. However, a more food-based dietary pattern may be of greater interest and is applied by recent guidelines. Reductions in the consumption of free sugar, added sugar, sugar-sweetened beverages are recommended to improve health [43]. Further, sucrose-rich diets impaired diabetes in animal models independently of total carbohydrate intake [44]. Every 10% increase in ultra-processed food consumption was associated with an 8% higher risk of developing IBS [45].

No significant association has been found between fat intake and increased weight in prospective studies [46,47]. In the current study, the reduced intake of carbohydrates, starch, and sucrose correlated with reduced weight and BMI, whereas changes in fat intake did not correlate. Accordingly, low-carbohydrate diets have shown beneficial effects on body weight in meta-analyses, which has not been described for low-fat diets [36,48]. However, another meta-analysis showed that low-carbohydrate, low-fat, and macronutrient diets induced reduced weight and systolic and diastolic blood pressure at 6 but not 12 months [49]. Thus, people may choose a diet they prefer. The obstacle is to maintain the weight reduction [49]. In comparison to a low-fat diet, low-carbohydrate diets improved HbA1c and fasting glucose levels, increased HDL cholesterol levels, and decreased body weight, according to a systemic review [50]. However, all changes returned to baseline after 2 years, highlighting the challenges in maintaining the changed dietary habits.

The high proportion of patients in the SSRD group who had decreased their intake of carbohydrates, starch, and sucrose suggests good compliance to the dietary advice. The low FODMAP group did not decrease these intakes to the same extent, which was shown both in the percentage of subjects with reduced intake and in the amount of intake. The relatively high proportion of controls who decreased their carbohydrate intake may be explained by the fact that they knew they were enrolled in a study examining reduced starch and sucrose intake as the active arm, and they could have made some changes according to this, although randomized to the control arm. The fat intake was higher in the second IBS cohort, with higher circulating lipid levels. One hypothesis behind these findings may be that the COVID-19 pandemic occurred between the two dietary interventions, and changes in lifestyle habits and weight were observed during this period [51]. Although the fat intake was slightly increased after SSRD, cholesterol levels were reduced in the second cohort with basically higher fat intake due to the changed composition of food and BMI reduction. Our previous study showed that the weight reduction was more pronounced after the SSRD than after low FODMAP [16]. The SSRD reduced the carbohydrate, starch, and sucrose intake more than the low FODMAP diet, which may explain the more aggravated reduction in weight in the SSRD group [16,17]. It is important not to increase the fat intake too much, since pronounced carbohydrate-restricted diets with markedly increased fat intake may lead to hyperlipidemia [9].

Homozygous carriers of an *SI* variant had markedly improved metabolic profiles but did not have a significantly different intake of energy or nutritional components, except for added sugar [11]. Much of the sugar and starch ingested in the Western world are unnecessary nutrients, generating high calorie intake with weight gain as a result. Sucrose and starch can be excluded from the diet without, or with only small, replacements with other food components. Thus, the very high fat intake in the LCHF diet is not necessary to reduce the adverse effects of sugar. This is shown in the present study, where protein and fat intake were only slightly increased.

There are several limitations of the study. One of the limitations is the merging of the two studies performed at two different time points. Although the same protocol was used, aspects in the environment may have changed among the general population, e.g., dietary habits and the range of goods available in the grocery store. These aspects could not be adjusted for in the statistical analyses. Another limitation of the study is that the diary registration differed between the cohorts, with written diaries in the first cohort and digitally reported ones in the second. However, even if there were small differences between the registrations, the participants were compared with themselves in the correlations and regression models. The control group was small, but the within-group changes in the active arms calibrate expectations. Since triglycerides could not be measured in the non-fasting blood samples, the metabolic syndrome could not be diagnosed. However, the indications of the syndrome were present with high waist circumference and low HDL cholesterol levels. The models were created to consider different aspects of influence, but collinearity may have weakened the associations. To compensate for this, correlations were also calculated, which showed less significant correlations than the model calculations, suggesting that the models with adjustment for several factors were more representative. The statistical models were rather rough, and more advanced models with complete inclusion of all nutrients and metabolomics are planned for a future study.

## 5. Conclusions

In conclusion, overweight/obesity was present in half of the IBS patients, with visceral adiposity in 60–70% of the patients and low HDL levels in about 25%. Weight and BMI were associated with lipid levels and symptoms at baseline. Both symptoms and weight were reduced after dietary interventions with an SSRD and low FODMAP diet. Reduced symptoms were more often associated with weight and BMI reduction during the study than with altered nutrient intake. Reduced sucrose intake was more often associated with reduced symptoms than reduced starch intake. These findings, together with those from previous cohort studies, systematic reviews, and meta-analyses [3,4,6], raise the hypothesis that the increased prevalence of overweight/obesity may be of major importance for the development and severity of IBS.

## Figures and Tables

**Table 1:** Basal characteristics.

Parameters	SSRD *n* = 157	Low FODMAP *n* = 78	Control *n* = 25	*p*-Value
**Age (year)**	45 (34–56)	43 (34–56)	35 (29–50)	0.441
**Gender (female) (*n*,%)**	122 (77.7)	68 (87.2)	22 (88.0)	0.172
**Disease duration (year)** *Missing*	18 (8–28) 11	20 (10–33) 3	11 (10–20) 6	0.448
**Education (*n*,%)** *Missing*	2	0	0	0.633
Primary school	8 (5.1)	2 (2.6)	2 (8.0)	
Secondary school	28 (17.8)	13 (16.7)	5 (20.0)	
Education after secondary school	40 (25.5)	17 (21.8)	3 (12.0)	
Examination at university	79 (50.3)	45 (57.7)	15 (60.0)	
**Occupation (*n*,%)** *Missing*	3	0	0	0.133
Working full time	93 (59.2)	45 (57.7)	8 (32.0)	
Working 99–51%	15 (9.6)	9 (11.5)	4 (16.0)	
Working 50%	5 (3.2)	1 (1.3)	2 (8.0)	
Studying	11 (7.0)	10 (12.8)	4 (16.0)	
Sick leave	7 (4.5)	2 (2.6)	1 (4.0)	
Unemployment	5 (3.2)	2 (2.6)	1 (4.0)	
Retirement	18 (11.5)	8 (10.3)	4 (16.0)	
Other				
**Marital status (*n*,%)** *Missing*	2	0	0	0.029
Living alone	46 (29.3)	14 (17.9)	11 (44.0)	
Living together	103 (65.6)	55 (70.5)	14 (56.0)	
Other	6 (3.8)	8 (10.3)		
**Smoking (*n*,%)** *Missing*	3	0	0	0.023
Never	84 (53.5)	42 (53.8)	12 (48.0)	
Former	56 (35.7)	28 (35.9)	7 (28.0)	
Present un regular	3 (1.9)	6 (7.7)	4 (16.0)	
Present regular	11 (7.0)	1 (1.3)	2 (8.0)	
**Alcohol intake for 1 week (standard glass) (*n*,%)** *Missing*	2	1	0	0.922
<1	66 (42.0)	33 (42.3)	14 (56.0)	
1–4	59 (37.6)	29 (37.2)	9 (36.0)	
5–9	23 (14.6)	13 (16.7)	2 (8.0)	
10–14	4 (2.5)	2 (2.6)	0	
≥15	3 (1.9)	0	0	
**Physical activity for 1 week (*n*,%)** *Missing*	2	1	0	0.834
No time	19 (12.1)	8 (10.3)	2 (8.0)	
<30 min	30 (19.1)	14 (17.9)	5 (20.0)	
30–60 min	28 (17.8)	14 (17.9)	4 (16.0)	
60–90 min	16 (10.2)	16 (20.5)	4 (16.0)	
90–120 min	20 (12.7)	8 (10.3)	2 (8.0)	
>120 min	42 (26.8)	17 (21.8)	8 (32.0)	

SSRD = starch- and sucrose-reduced diet; FODMAP = fermentable oligo-, di-, and monosaccharides and polyols. Kruskal–Wallis test or Fisher’s exact test. Values are given as median (interquartile ranges) or number (percentages). *p* < 0.05 was considered statistically significant.

**Table 2:** Nutrient intake before and after the dietary intervention.

	SSRD *n* = 157		Low FODMAP *n* = 78		Control *n* = 25		*p*-Value *
Parameters	Median (IQR)	*p*-Value	Median (IQR)	*p*-Value	Median (IQR)	*p*-Value	
**Energy** (kcal)							
Baseline	1759 (1446–2108)		1800 (1475–2027)		1387 (1202–2027)		0.183
4 weeks	1498 (1152–1911)	<0.001	1664 (1303–2049)	0.024	1640 (1216–2168)	0.424	0.346
**Carbohydrates** (g)							
Baseline	182 (145–218)		178 (144–212)		177 (112–207)		0.833
4 weeks	86 (66–118)	<0.001	152 (114–184)	<0.001	182 (89–224)	0.235	<0.001
**Protein** (g)							
Baseline	72 (54–82)		66 (52–80)		59 (46–71)		0.066
4 weeks	80 (59–97)	<0.001	68 (51–81)	0.471	65 (53–81)	0.571	0.193
**Fat** (g)							
Baseline	70 (54–93)		73 (54–93)		61 (46–72)		0.058
4 weeks	81 (60–107)	0.013	76 (52–93)	0.308	69 (46–96)	0.566	0.072
**Saturated (g)**							
Baseline	26 (18–35)		29 (21–35)		20 (17–31)		0.179
4 weeks	25 (18–35)	0.427	25 (18–32)	0.067	25 (14–39)	0.925	0.198
**Mono-unsat** (g)							
Baseline	29 (20–37)		29 (21–40)		22 (17–30)		0.043
4 weeks	32 (22–43)	0.022	30 (21–41)	0.629	25 (16–35)	0.696	0.201
**Poly-unsat** (g)							
Baseline	11 (7–14)		11 (8–15)		8 (6–10)		0.018
4 weeks	13 (9–19)	<0.001	11 (7–15)	0.736	9 (7–12)	0.058	0.031
**Fiber** (g)							
Baseline	18 (13–24)		18 (15–22)		16 (12–22)		0.674
4 weeks	17 (12–22)	0.040	15 (11–20)	0.001	15 (11–22)	0.793	0.307
**Starch** (g)							
Baseline	58 (35–83)		46 (35–65)		71 (43–90)		0.009
4 weeks	17 (4–38)	<0.001	42 (30–63)	0.768	82 (37–101)	0.849	<0.001
**Sucrose** (g)							
Baseline	24 (13–38)		26 (15–41)		20 (13–43)		0.429
4 weeks	6 (3–10)	<0.001	18 (12–36)	0.027	19 (5–36)	0.144	<0.001

There were 2 missing values in the starch- and sucrose-reduced diet (SSRD) group and 2 in the low content of fermentable oligo-, di-, and monosaccharides and polyols (FODMAP) group at baseline. After 4 weeks, there were 15 missing values for SSRD, 10 for low FODMAP, and 3 for controls. Kruskal–Wallis test * for comparisons between groups of baseline values and differences from 4 weeks and baseline and Wilcoxon Signed Ranks for comparisons within groups. Values are given as median (interquartile ranges). *p* < 0.05 was considered statistically significant.

**Table 3:** Correlations between weight and BMI and nutrient intake.

	Weight (kg)	BMI (kg/m^2^)
**Baseline**		
Energy (kcal)	Rs = 0.171, *p* = 0.007, *q* = 0.028	Rs = 0.116, *p* = 0.065, *q* = 0.201
Carbohydrates (g)	Rs = 0.148, *p* = 0.018, *q* = 0.042	Rs = 0.109, *p* = 0.086, *q* = 0.201
Protein (g)	Rs = 0.135, *p* = 0.032, *q* = 0.056	Rs = 0.045, *p* = 0.481, *q* = 0.561
Fat (g)	Rs = 0.167, *p* = 0.008, *q* = 0.028	Rs = 0.128, *p* = 0.042, *q* = 0.201
Fiber (g)	Rs = 0.005, *p* = 0.937, *q* = 0.937	Rs = (−0.032), *p* = 0.618, *q* = 0.618
Starch (g)	Rs = 0.018, *p* = 0.782, *q* = 0.937	Rs = (−0.065), *p* = 0.306, *q* = 0.444
Sucrose (g)	Rs = 0.007, *p* = 0.917, *q* = 0.937	Rs = 0.063, *p* = 0.317, *q* = 0.444
**Differences (** **∆)**	**∆ Weight (kg)**	**∆ BMI (kg/m^2^)**
Energy (kcal)	Rs = 0.170, *p* = 0.011, *q* = 0.026	Rs = 0.182, *p* = 0.006, *q* = 0.014
Carbohydrates (g)	Rs = 0.254, *p* < 0.001, *q* = 0.004	Rs = 0.262, *p* < 0.001, *q* = 0.004
Protein (g)	Rs = 0.086, *p* = 0.198, *q* = 0.277	Rs = 0.090, *p* = 0.179, *q* = 0.251
Fat (g)	Rs = 0.044, *p* = 0.515, *q* = 0.601	Rs = 0.051, *p* = 0.452, *q* = 0.527
Fiber (g)	Rs = (−0.026), *p* = 0.694, *q* = 0.694	Rs = (−0.023), *p* = 0.727, *q* = 0.727
Starch (g)	Rs = 0.224, *p* < 0.001, *q* = 0.004	Rs = 0.231, *p* < 0.001, *q* = 0.004
Sucrose (g)	Rs = 0.155, *p* = 0.020, *q* = 0.035	Rs = 0.160, *p* = 0.016, *q* = 0.028

BMI = body mass index. There were 8 missing calculations at baseline and 36 missing calculations in the differences. Spearman’s correlation test. *p*-values were adjusted for false discovery rate (FDR) set at 5% according to the Benjamini–Hochberg method [33]. The FDR-adjusted *q*-values were the main result. *q* < 0.05 was considered statistically significant.

**Table 4:** Gastrointestinal symptoms before and after the dietary intervention.

	SSRD *n* = 157		Low FODMAP *n* = 78		Control *n* = 25		*p*-Value *
Parameters	Median (IQR)	*p*-Value	Median (IQR)	*p*-Value	Median (IQR)	*p*-Value	
**Abdominal Pain** 5 (1–13)							
Baseline	49 (34–64)		50 (32–65)		49 (27–62)		0.936
4 weeks	19 (4–37)	<0.001	13 (0–27)	<0.001	50 (32–63)	0.650	<0.001
**Diarrhea** 3 (0–10)							
Baseline	54 (19–74)		37 (4–74)		47 (5–70)		0.250
4 weeks	15 (2–34)	<0.001	8 (0–24)	<0.001	24 (1–49)	0.300	0.067
**Constipation** 6 (2–16)							
Baseline	50 (2–72)		54 (10–76)		54 (30–69)		0.581
4 weeks	16 (1–40)	<0.001	21 (0–55)	<0.001	28 (1–68)	0.045	0.749
**Bloating and flatulence** 10 (2–23)							
Baseline	75 (58–86)		73 (54–86)		78 (68–89)		0.406
4 weeks	26 (11–54)	<0.001	19 (8–50)	<0.001	69 (56–80)	0.001	<0.001
**Vomiting and nausea** 2 (0–4)							
Baseline	12 (2–34)		13 (1–36)		29 (6–50)		0.210
4 weeks	3 (0–16)	<0.001	0 (0–11)	<0.001	12 (2–56)	0.112	0.742
**Intestinal symptoms’ influence on daily life** 2 (0–14)							
Baseline	73 (55–85)		70 (54–84)		68 (53–78)		0.718
4 weeks	30 (14–62)	<0.001	22 (10–50)	<0.001	65 (51–82)	0.732	<0.001
**Psychological well-being** 5 (2–15)							
Baseline	46 (18–66)		45 (16–59)		47 (24–71)		0.396
4 weeks	24 (9–51)	<0.001	18 (2–34)	<0.001	48 (32–60)	0.732	<0.032
**Total IBS-SSS**							
Baseline	302 (239–352)		300 (238–360)		310 (247–351)		0.944
4 weeks	135 (78–233)	<0.001	116 (63–176)	<0.001	300 (233–331)	0.248	<0.001

There were 2 missing values in the starch- and sucrose-reduced diet (SSRD) group and 1 in the low content of fermentable oligo-, di-, and monosaccharides and polyols (FODMAP) group at baseline. After 4 weeks, there were 11 missing values for SSRD, 7 for low FODMAP, and 2 for controls. Specific gastrointestinal symptoms were assessed using the visual analog scale for irritable bowel syndrome (VAS-IBS) and total gastrointestinal symptoms were assessed by the irritable bowel syndrome severity scoring system (IBS-SSS) [20,30]. Kruskal–Wallis test * for comparisons between groups of baseline values and differences from 4 weeks and baseline and Wilcoxon Signed Ranks for comparisons within groups. Values are given as median (interquartile ranges). *p* < 0.05 was considered statistically significant.

**Table 5:** Extraintestinal symptoms before and after the dietary intervention.

	SSRD *n* = 157		Low FODMAP *n* = 78		Control *n* = 25		*p*-Value *
Parameters	Median (IQR)	*p*-Value	Median (IQR)	*p*-Value	Median (IQR)	*p*-Value	
**Difficulties eating a meal**							
Baseline	11 (2–29)		6 (0–22)		9 (1–26)		0.315
4 weeks	2 (0–13)	<0.001	0 (0–9)	<0.001	6 (1–20)	0.071	0.544
**Headache**							
Baseline	29 (7–66)		27 (9–58)		31 (15–48)		0.934
4 weeks	15 (4–35)	<0.001	12 (0–35)	<0.001	25 (8–35)	0.845	0.061
**Back pain**							
Baseline	29 (6–68)		28 (4–65)		45 (19–66)		0.419
4 weeks	10 (0–34)	<0.001	4 (0–35)	<0.001	32 (5–55)	0.249	0.395
**Fatigue**							
Baseline	60 (33–84)		74 (48–84)		67 (39–91)		0.386
4 weeks	36 (17–67)	<0.001	37 (14–60)	<0.001	65 (41–83)	0.794	0.002
**Belching/excess wind**							
Baseline	71 (48–85)		75 (52–87)		67 (20–84)		0.430
4 weeks	22 (8–42)	<0.001	21 (8–45)	<0.001	59 (27–78)	0.246	<0.001
**Reflux**							
Baseline	19 (5–51)		20 (2–60)		17 (2–78)		0.979
4 weeks	4 (0–20)	<0.001	3 (0–26)	<0.001	8 (1–50)	0.061	0.132
**Urinary urgency**							
Baseline	28 (4–70)		22 (4–64)		20 (3–56)		0.603
4 weeks	10 (0–35)	<0.001	3 (0–22)	<0.001	16 (1–43)	0.131	0.091
**Leg pain**							
Baseline	2 (0–15)		0 (0–18)		3 (0–16)		0.489
4 weeks	1 (0–11)	<0.001	0 (0–5)	<0.001	4 (1–12)	0.924	0.282
**Muscle/joint pain**							
Baseline	34 (6–66)		30 (4–72)		21 (9–70)		0.934
4 weeks	18 (2–44)	<0.001	12 (0–39)	<0.001	49 (9–75)	0.753	0.037
**Total extraintestinal** **IBS-SSS**							
Baseline	174 (119–232)		172 (120–242)		197 (106–257)		0.948
4 weeks	96 (39–160)	<0.001	77 (44–136)	<0.001	169 (107–208)	0.231	<0.001

There were 2 missing values in the starch- and sucrose-reduced diet (SSRD) group and 1 in the low content of fermentable oligo-, di-, and monosaccharides and polyols (FODMAP) group at baseline. After 4 weeks, there were 11 missing values for SSRD, 7 for low FODMAP, and 2 for controls. Specific and total extraintestinal symptoms were assessed by the irritable bowel syndrome severity scoring system (IBS-SSS) [20]. Kruskal–Wallis test * for comparisons between groups of baseline values and differences from 4 weeks and baseline and Wilcoxon Signed Ranks for comparisons within groups. Values are given as median (interquartile ranges). *p* < 0.05 was considered statistically significant.

**Table 6:** Cholesterol values before and after the dietary intervention.

	**SSRD 2018** ***n* = 80**		**SSRD 2022** ***n* = 77**		**Low FODMAP** ***n* = 78**		**Control** ***n* = 25**		***p*-** Value *
**Lipids**	Median (IQR)	*p*	Median (IQR)	*p*	Median (IQR)	*p*	Median (IQR)	*p*	
**Cholesterol ** (3.3–6.9 mmol/L) *Missing baseline*, *4 weeks*	1, 11		2, 7		1, 6		0, 2		
Baseline	4.60 (4.00–5.20)		5.00 (4.40–5.60)		4.70 (4.35–5.85)		4.80 (4.10–5.70)		0.079
4 weeks	4.70 (4.25–5.20)	0.856	4.80 (4.00–5.40)	0.007	4.90 (4.20–5.48)	0.003	4.80 (4.00–5.80)	0.805	0.066
**HDL** (1.0–2.7 mmol/L) *Missing baseline*, *4 weeks*	1, 11		2, 7		1, 6		0, 2		
Baseline	1.40 (1.20–1.60)		1.50 (1.20–1.80)		1.60 (1.25–1.80)		1.40 (1.20–1.70)		0.002
4 weeks	1.40 (1.20–1.60)	0.393	1.45 (1.20–1.90)	0.185	1.60 (1.32–1.80)	0.446	1.40 (1.20–1.70)	0.013	0.080
**LDL** (1.4–4.7 mmol/L) *Missing baseline*, *4 weeks*	1, 11		2, 7		1, 6		0, 2		
Baseline	2.40 (1.80–2.80)		3.20 (2.70–3.80)		3.10 (2.70–3.80)		2.70 (1.90–3.45)		<0.001
4 weeks	2.40 (1.90–2.70)	0.395	3.05 (2.38–3.72)	0.004	3.05 (2.60–3.60)	0.036	2.20 (1.90–3.20)	0.365	0.063
**Non-HDL** (1.9–5.1 mmol/L) *Missing baseline*, *4 weeks*	1, 11		6, 7		5, 6		0, 2		
Baseline	3.10 (2.70–3.80)		3.40 (2.80–4.00)		3.30 (2.85–4.10)		3.20 (2.70–4.30)		0.638
4 weeks	3.40 (2.75–3.60)	0.848	3.30 (2.60–3.80)	0.015	3.20 (2.72–3.88)	0.002	3.20 (2.60–4.30)	0.340	0.166

SSRD = starch- and sucrose-reduced diet; FODMAP = fermentable oligo-, di-, and monosaccharides and polyols; HDL= high-density lipoprotein; LDL = low-density lipoprotein. Reference values within brackets from the Department of Clinical Chemistry [32]. Kruskal–Wallis test * was used for comparisons between the four groups SSRD 2018, SSRD 2022, low FODMAP, and controls. Wilcoxon Signed Ranks was used for comparisons within groups. Values are given as median (interquartile ranges). *p* < 0.05 was considered statistically significant.

## Data Availability

The data presented in this study are available on request from the corresponding author due to ethical reasons.

## Supplementary Materials

The following supporting information can be downloaded at https://www.mdpi.com/article/10.3390/nu17182966/s1: Supplementary Figure S1: CONSORT flow chart over RCT 1; Supplementary Figure S2: CONSORT flow chart over RCT 1; Supplementary Figure S3: Hypothesis behind improvements in symptoms and lipid levels after dietary interventions; Table S1: Recommendations of fruit intake according to a starch- and sucrose-reduced diet; Table S2: Recommendations of vegetable and legume intake according to a starch- and sucrose-reduced diet; Table S3: Comorbidity and drug use in irritable bowel syndrome; Table S4: Nutrient intake and lipid values at baseline stratified according to the two study cohorts; Table S5: Weight and BMI changes; Table S6: Correlations at baseline between weight, BMI, and nutrient intake and gastrointestinal symptoms; Table S7: Correlations between differences (∆) in weight, BMI, and nutrient intake and gastrointestinal symptoms during the study; Table S8: Correlations at baseline between weight, BMI, and nutrient intake and extraintestinal symptoms; Table S9: Correlations between differences (**∆**) in weight, BMI, and nutrient intake and extraintestinal symptoms during the study; Table S10: Correlations between weight/BMI and nutrients and lipid values; Table S11: Associations between weight and nutrient intake, and lipid levels and symptoms at baseline; Table S12: Associations between differences (∆) in weight and nutrient intake, and lipid levels and symptoms: Table S13: Significant associations between BMI and nutrient intake, and lipid levels and symptoms at baseline; Table S14: Significant associations between differences (∆) in weight and nutrient intake, and lipid levels and symptoms; Table S15: Associations between BMI, sucrose, and starch, and lipid levels and symptoms at baseline; Table S16: Associations between differences (∆) in body mass index (BMI), sucrose, and starch, and lipid levels and symptoms.

## Abbreviations

The following abbreviations are used in this manuscript:

IBS	Irritable bowel syndrome
FODMAP	Fermentable oligo-, di-, and monosaccharides and polyols
SSRD	Starch- and sucrose-reduced diet
