# Supplementary material for: Dietary Modifications in IBS Leads to Reduced Symptoms, Weight, and Lipid Levels: Two Randomized Clinical Trials
Source: Nutrients. 2025 Sep 16;17(18):2966. doi: 10.3390/nu17182966 (PMC12472658; doi:10.3390/nu17182966)
Supplement: Supplementary file 1 [file nutrients-17-02966-s001.zip › nutrients-3866867-supplementary.pdf]

Supplementary Figure S1. CONSORT 2025 flow diagram over RCT 1 (2018-2019).

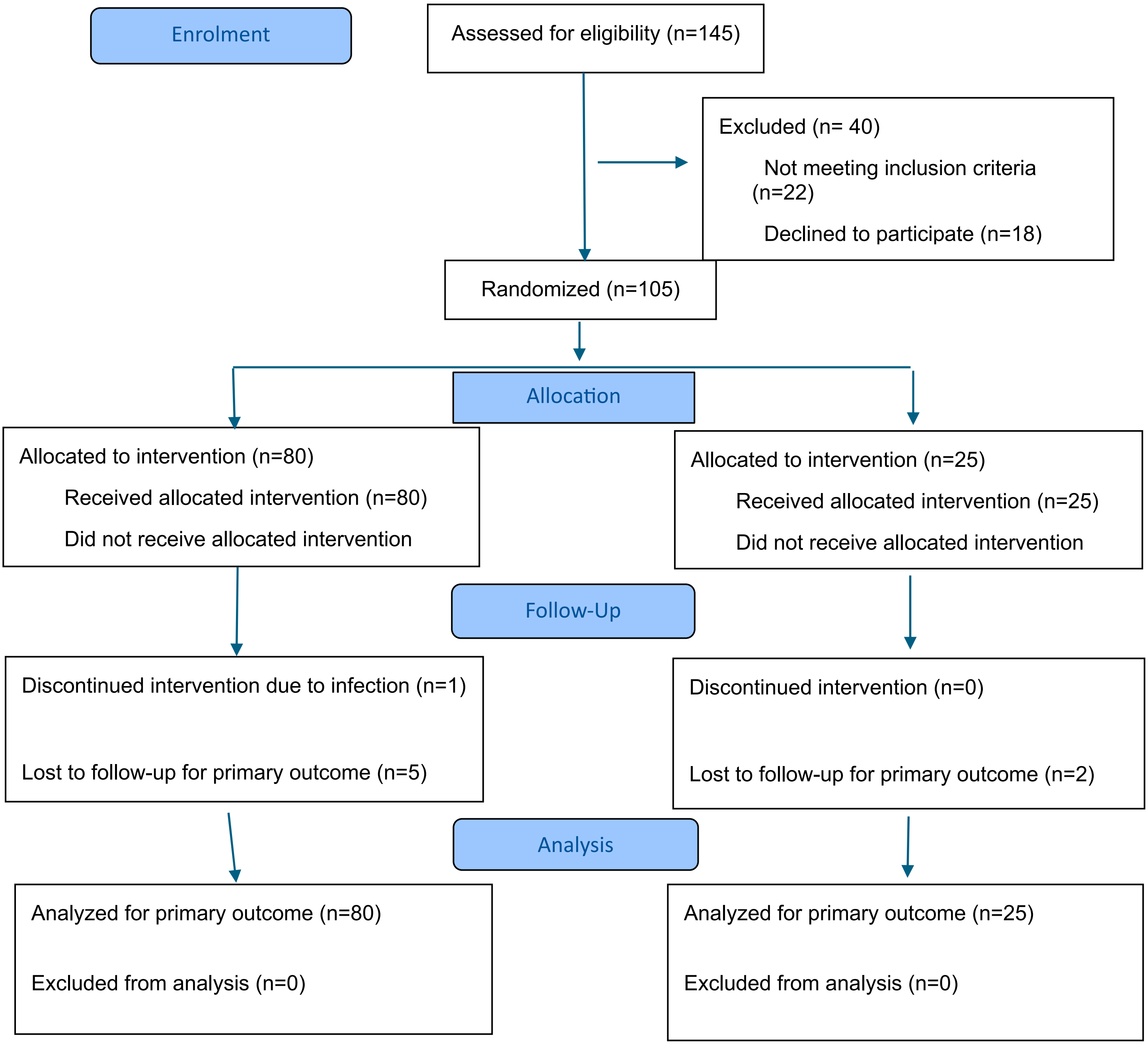

Supplementary Figure S2. CONSORT 2025 flow diagram over RCT 2 (2022-2024).

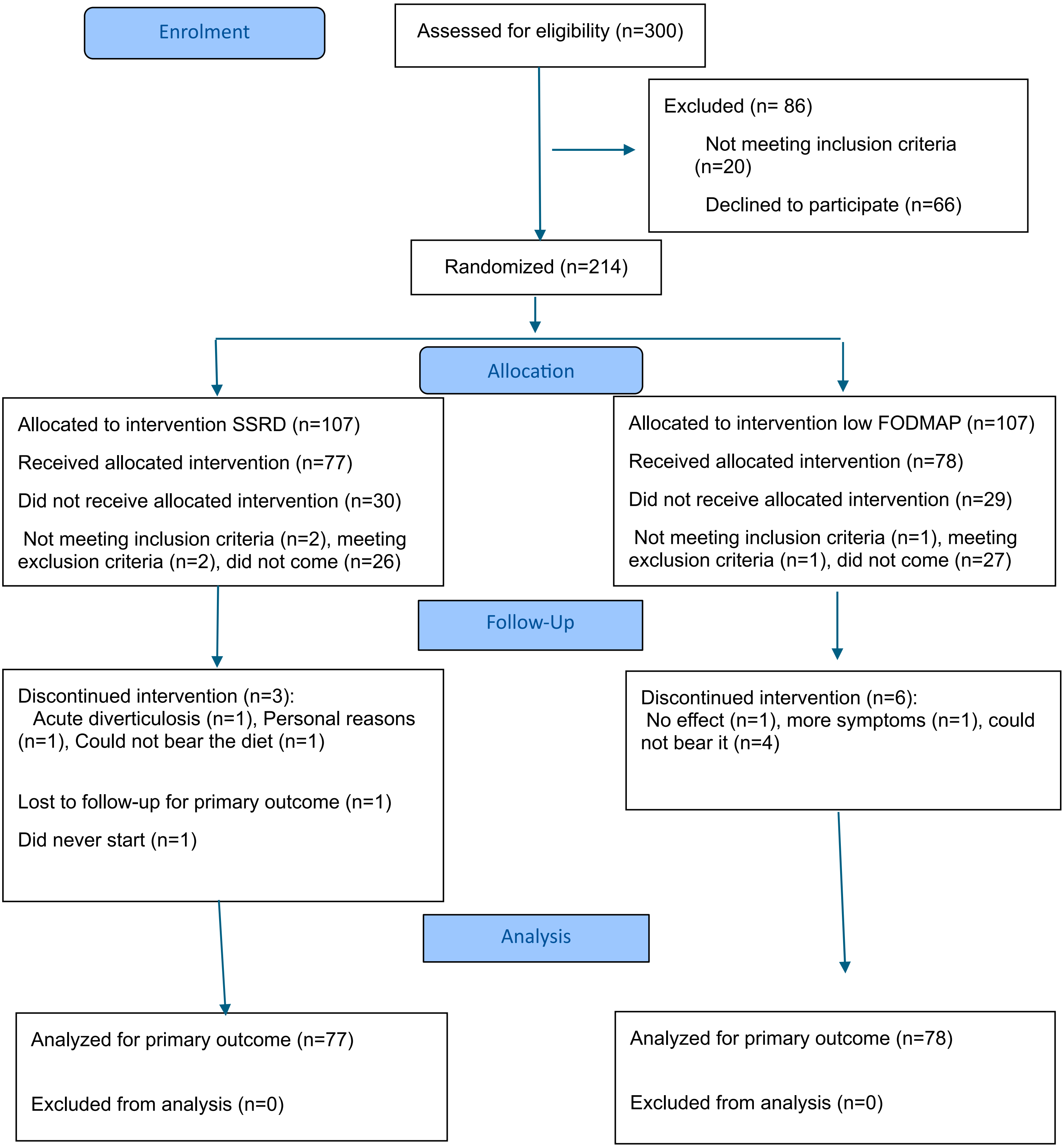

Supplementary Figure S3. Hypothesis behind improvements of symptoms and lipid levels after dietary interventions. FODMAP = fermentable oligo-, di-, and monosaccharides and polyols.

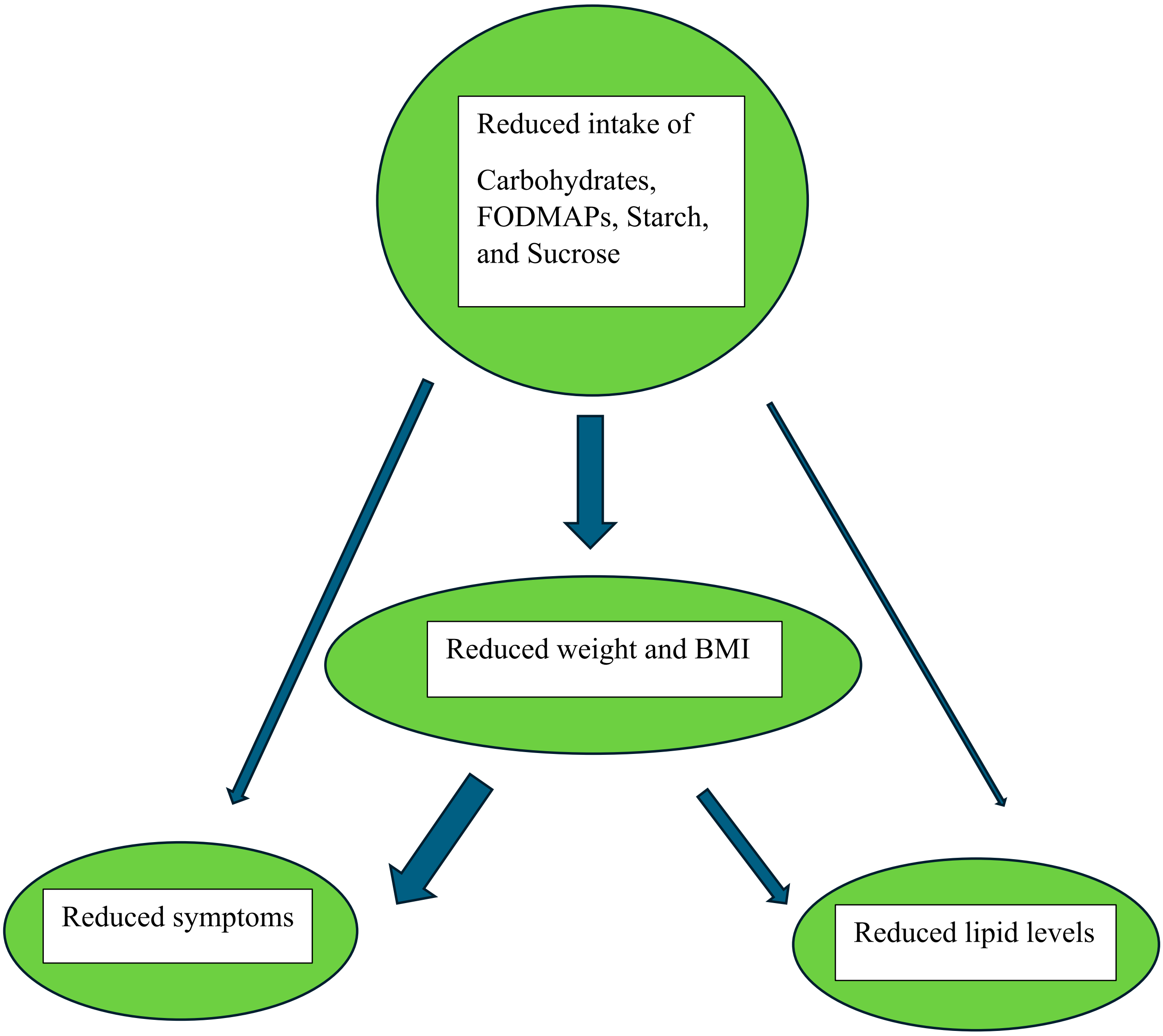

Supplementary Table S1. Recommendations of fruit intake according to a starch- and sucrose-reduced diet.

**Well tolerated**	**Tolerated by some**	**Not tolerated**
Avocado	Persimmons	Apples
Blackberries	Plums	Apricots
Blueberries	Raisins	Bananas
Boysenberries	Watermelon	Cantaloupe
Cherries		Dates
Cranberries		Grapefruit
Currants		Guava
Figs		Honeydew melon
Gooseberries		Mangos
Grapes		Nectarines
Kiwi		Oranges
Lemons		Peaches
Limes		Pineapple
Loganberries		Tangelos
Olives		Tangerines
Papaya		
Pears		
Pomegranates		
Prunes		
Raspberries		
Rhubarb		
Strawberries		

Supplementary Table S2. Recommendations of vegetable and legume intake according to a starch- and sucrose-reduced diet.

**Well tolerated**	**Tolerated by some**	**Not tolerated**
Alfalfa sprouts	Edamame soybeans	Beets
Artichokes*	Jicamas	Black beans
Arugulas	Leeks	Black-eyed peas
Asparagus*	Okra	Butternut
Bamboo shoots	Pumpkin	Carrots
Bok choy	Snow peas	Cassava
Broccoli*	Tempeh	Chickpeas
Brussel sprouts*	Tofu	Corn
Cabbage*	Yellow wax beans	Garlic
Cauliflower*		Green peas
Celery		Lentils
Chard		Kidney beans
Chicories		Lima beans
Chives		Navy beans
Collard greens		Onion
Cress		Parsnips
Cucumber		Pinto beans
Eggplant		Potatoes
Endive		Soybeans
Green beans		Split peas
Kale		Sweet potatoes
Lettuce		Yams
Mung bean sprouts		
Mushrooms		
Mustard green		
Peppers		
Radishes		
Spaghetti squash		
Spinach		
Tomatoes		
Turnips		
Yellow squash		
Zucchini		

* Excess intake can cause bloating/flatulence in all individuals.

Supplementary Table S3. Comorbidity and drug use in irritable bowel syndrome.

**Comorbidity**	**IBS 2018 N=105**	**IBS2022 N=155**	**p-values**
Allergy	17 (16.2)	17 (11.0)	0.262
Anxiety	3 (2.9)	7 (4.5)	0.744
Asthma bronchialis	11 (10.5)	7 (4.5)	0.081
Burned out	5 (4.8)	10 (6.5)	0.787
Depression	11 (10.5)	10 (6.5)	0.255
Eczema	5 (4.8)	19 (12.3)	0.049
Fibromyalgia	3 (2.9)	9 (5.8)	0.371
Hypertension	10 (9.5)	12 (7.7)	0.654
Hypothyroid disease	12 (11.4)	6 (3.9)	0.024
Lactose intolerance	5 (4.8)	10 (6.5)	0.787
Migraine/headache	7 (6.7)	10 (6.5)	1.00
Reflux/hiatushernia	5 (4.8)	18 (11.6)	0.074
**Drug treatment**			
Allergy medicines	11 (10.5)	24 (15.5)	0.272
Antidepressants	18 (17.1)	23 (14.8)	0.609
Asthma inhalators	6 (5.7)	8 (5.2)	1.00
Hormonal treatment*	8 (7.6)	24 (15.5)	0.082
Laxatives/bulking agents	14 (13.3)	38 (24.5)	0.028
Levaxine	13 (12.4)	6 (3.9)	0.014
NSAID	41 (39.0)	50 (32.3)	0.290
Paracetamols	45 (42.9)	54 (34.8)	0.196
Proton pump inhibitors	16 (15.2)	48 (31.0)	0.005
**Dietary supplements**			
Iron	1 (1.0)	7 (4.5)	0.148
Other minerals (Ca, Mg, Z, Si)	9 (8.6)	26 (16.8)	0.065
Vitamin B/Folic acid	9 (8.6)	14 (9.0)	1.00
Vitamin D	14 (13.3)	26 (16.8)	0.448
Multivitamins	7 (6.7)	23 (14.8)	0.049
Vitamin D and/or multivitamins	16 (15.2)	39 (25.2)	0.064
Probiotics	19 (18.1)	15 (9.7)	0.061

IBS = irritable bowel syndrome. * = combination pills. Values are given as number (percentage). Differences were calculated between the two IBS cohorts by Fisher's exact test. $p<0.05$ was considered statistically significant.

Supplementary Table S4. Nutrient intake and lipid values at baseline stratified according to the two study cohorts.

	**IBS 2018**<br>**N = 105**	**IBS2022**<br>**N = 155**	**p-values**
Nutrient intake			
**Energy (kcal)**	1609 (1315-269)	1791 (1490-2074)	0.120
**Carbohydrates (g)**	184 (137-221)	180 (145-207)	0.479
**Protein (g)**	67 (50-81)	69 (53-81)	0.768
**Fat (g)**	62 (45-91)	75 (57-93)	0.004
**Saturated (g)**	24 (15-36)	27 (21-34)	0.040
**Mono-unsat (g)**	24 (16-34)	30 (22-40)	<0.001
**Polyunsat (g)**	9 (6-13)	11 (8-15)	0.004
**Fiber (g)**	18 (13-26)	18 (14-22)	0.884
**Sucrose (g)**	23 (13-38)	25 (14-40)	0.327
**Starch (g)**	76 (48-111)	45 (31-63)	<0.001
Lipid values			
**Cholesterol (mmol/L)**	4.60 (4.00-5.28)	4.90 (4.40-5.70)	0.013
**HDL (mmol/L)**	1.40 (1.20-1.60)	1.60 (1.20-1.80)	<0.001
**LDL (mmol/L)**	2.40 (1.82-2.88)	3.20 (2.70-3.80)	<0.001
**Non-HDL (mmol/L)**	3.15 (2.70-3.80)	3.30 (2.80-4.00)	0.267

HDL = high-density lipoprotein; LDL = low-density lipoprotein; IBS = irritable bowel syndrome. There were 2 missing values in each cohort. Mann–Whitney U test for comparison between cohorts at baseline. Values are given as median (interquartile ranges). p<0.05 was considered statistically significant.

Supplementary Table S5. Weight and BMI changes.

	**SSRD N = 157**		**Low FODMAP N = 78**		**Control N = 25**		**p-Value***
Parameters	Median (IQR)	p-Value	Median (IQR)	p-Value	Median (IQR)	p-Value	
**Weight (kg)**							
Baseline *Missing*	72(64-83) 5		69(63-83) 0		68(57-75) 1		0.094
4 weeks *Missing*	71(64-81) 15	<0.001	68(62-83) 6	<0.001	68(61-76) 3	0.158	<0.001
**BMI (kg/m^2)**							
Baseline *Missing*	25(23-28) 5		25(22-28) 0		24(21-26) 1		<0.001
4 weeks *Missing*	25(22-28) 15	<0.001	25(22-27) 6	<0.001	25(22-26) 3	0.273	0.999

BMI = body mass index; SSRD = starch- and sucrose-reduced diet; FODMAP = fermentable oligo-, di-, and monosaccharides and polyols. Kruskal–Wallis test * for comparison between groups and Wilcoxon Signed Ranks for comparisons within groups. Values are given as median (interquartile ranges). p<0.05 was considered statistically significant.

Supplementary Table S6. Correlations at baseline between weight, BMI, and nutrient intake and gastrointestinal symptoms.

Baseline	**Weight**	**BMI**	**Energy**	**Carbohydrates**	**Protein**	**Fat**	**Fiber**	**Sucrose**	**Starch**
**Abdominal pain**	Rs=(-0.116), p=0.066, q=0.106	Rs=(-0.076), p=0.228	Rs=(-00.049), p=0.440	Rs=(-0.116), p=0.065, q=0.260	Rs=(-0.048), p=0.449	Rs=0.015, p=0.813	Rs=(-0.103), p=0.101, q=0.202	Rs=(-0.035), p=0.577	Rs=(-0.048), p=0.446
**Diarrhea**	Rs=0.054, p=0.396, q=0.528	Rs=0.017, p=0.793	Rs=(-0.047), p=0.454	Rs=(-0.075), p=0.235, q=0.614	Rs=(-0.048), p=0.448	Rs=(-0.008), p=0.903	Rs=(-0.140), p=0.025, q=0.100	Rs=(-0.080), p=0.206	Rs=(-0.079), p=0.209
**Constipation**	Rs=(-0.229), p<0.001, q=0.008	Rs=(-0.119), p=0.058	Rs=(-0.021), p=0.735	Rs=0.030, p=0.633, q=0.715	Rs=(-0.039), p=0.537	Rs=(-0.049), p=0.439	Rs=0.041, p=0.517, q=0.591	Rs=0.105, p=0.095	Rs=(-0.035), p=0.579
**Bloating and flatulence**	Rs=(-0.119), p=0.060, q=0.106	Rs=(-0.078), p=0.217	Rs=0.002, p=0.970	Rs=0.023, p=0.715, q=0.715	Rs=(-0.083), p=0.190	Rs=(-0.010), p=0.878	Rs=0.001, p=0.986, q=0.986	Rs=0.067, p=0.286	Rs=0.035, p=0.579
**Vomiting and nausea**	Rs=(-0.018), p=0.820, q=0.820	Rs=(-0.005), p=0.948	Rs=0.051, p=0.536	Rs=0.179, p=0.027, q=0.216,	Rs=0.015, p=0.854	Rs=(-0.004), p=0.960	Rs=0.208, p=0.010, q=0.080	Rs=0.148, p=0.069	Rs=0.028, p=0.734
**Influence on daily life**	Rs=(-0.156), p=0.013, q=0.052	Rs=(-0.112), p=0.076	Rs=(-0.055), p=0.384	Rs=(-0.041), p=0.511, q=0.715	Rs=(-0.112), p=0.075	Rs=(-0.017), p=0.792	Rs=(-0.077), p=0.223, q=0.357	Rs=(-0.029), p=0.640	Rs=(-0.071), p=0.261
**Psychological well-being**	Rs=(-0.046), p=0.470, q=0.537	Rs=(-0.062), p=0.324	Rs=(-0.034), p=0.587	Rs=0.064, p=0.307, q=0.614	Rs=(-0.046), p=0.468	Rs=(-0.092), p=0.144	Rs=(-0.116), p=0.065, q=0.173	Rs=0.067, p=0.288	Rs=0.039, p=0.531
**Total IBS-SSS**	Rs=(-0.137), p=0.030, q=0.080	Rs=(-0.073), p=0.249	Rs=(-0.022), p=0.725	Rs=(-0.037), p=0.553, q=0.715	Rs=(-0.068), p=0.279	Rs=0.010, p=0.876	Rs=(-0.057), p=0.368, q=0.491	Rs=0.002, p=0.969	Rs=(-0.031), p=0.622

BMI = body mass index. Specific and total gastrointestinal symptoms were assessed by the irritable bowel syndrome severity scoring system (IBS-SSS) and visual analog scale for irritable bowel syndrome (VAS-IBS) [20, 30]. There were as most 8 missing calculations. Spearman's correlation test. p-values were adjusted for false discovery rate (FDR) set at 5% according to the Benjamini–Hochberg method [33]. The FDR-adjusted q-values was the main result. q < 0.05 was considered statistically significant.

Supplementary Table S7. Correlations between differences (Δ) of weight, BMI, and nutrient intake and gastrointestinal symptoms during the study.

Differences (Δ)	**Δ Weight**	**Δ BMI**	**Δ Energy**	**ΔCarbohydrates**	**Δ Protein**	**Δ Fat**	**Δ Fiber**	**Δ Sucrose**	**Δ Starch**
**Abdominal pain**	Rs=0.005 p=0.935 q=0.935	Rs=0.002 p=0.976 q=0.976	Rs=0.064, p=0.329	Rs=0.072, p=0.274, q=0.548	Rs=(-0.039), p=0.558	Rs=0.043, p=0.514	Rs=0.086, p=0.194, q=0.376	Rs=0.015, p=0.823	Rs=(-0.046), p=0.488
**Diarrhea**	Rs=0.009, p=0.888 q=0.935	Rs=0.002, p=0.973 q=0.976	Rs=0.012, p=0.861	Rs=0.036, p=0.588, q=0.800	Rs=(-0.023), p=0.726	Rs=0.015, p=0.818	R=0.088, p=0.181, q=0.376	Rs=0.008, p=0.901	Rs=0.015, p=0.823
**Constipation**	Rs=0.039, p=0.552 q=0.935	Rs=0.049, p=0.461 q=0.922	Rs=0.050, p=0.447	Rs=0.034, p=0.611, q=0.800	Rs=0.119, p=0.071	Rs=(-0.021), p=0.749	Rs=(-0.008), p=0.901, q=0.901	Rs=(-0.002), p=0.979	Rs=(-0.004), p=0.455
**Bloating and flatulence**	Rs=0.133, p=0.043 q=0.172	Rs=0.140, p=0.033 q=0.132	Rs=0.113, p=0.088	Rs=0.175, p=0.008, q=0.032	Rs=0.005, p=0.941	Rs=0.038, p=0.564	Rs=0.146, p=0.027, q=0.168	Rs=0.067, p=0.309	Rs=0.066, p=0.318
**Vomiting and nausea**	Rs=0.008, p=0.928 q=0.935	Rs=0.010, p=0.903 q=0.976	Rs=0.019, p=0.826	Rs=0.013, p=0.878, q=0.878	Rs=0.015, p=0.860	Rs=(-0.023), p=0.794	Rs=0.051, p=0.559, q=0.649	Rs=0.017, p=0.848	Rs=(-0.138), p=0.112
**Influence on daily life**	Rs=0.140, p=0.034 q=0.172	Rs=0.143, p=0.030 q=0.132	Rs=0.076, p=0.248	Rs=0.130, p=0.049, q=0.131	Rs=(-0.049), p=0.461	Rs=0.040, p=0.548	Rs=0.079, p=0.235, q=0.376	Rs=0.056, p=0.397	Rs=0.046, p=0.484
**Psychological well-being**	Rs=0.006, p=0.923 q=0.935	Rs=0.003, p=0.962 q=0.976	Rs=(-0.056), p=0.397	Rs=0.025, p=0.700, q=0.800	Rs=(-0.044), p=0.501	Rs=(-0.055), p=0.403	Rs=(-0.038), p=0.568, q=0.649	Rs=0.013, p=0.846	Rs=0.006, p=0.929
**Total IBS-SSS**	Rs=0.113, p=0.086 q=0.299	Rs=0.109, p=0.100 q=0.267	Rs=0.121, p=0.066	Rs=0.182, p=0.006, q=0.032	Rs=(-0.008) p=0.907	Rs=0.052, p=0.434	Rs=0.134, p=0.042, q=0.168	Rs=0.118, p=0.073	Rs=0.054, p=0.418

BMI = body mass index. Specific and total gastrointestinal symptoms were assessed by the irritable bowel syndrome severity scoring system (IBS-SSS) and visual analog scale for irritable bowel syndrome (VAS-IBS) [20, 30]. There were 36 missing calculations. Spearman's correlation test. p-values were adjusted for false discovery rate (FDR) set at 5% according to the Benjamini–Hochberg method [33]. The FDR-adjusted q-values was the main result. $q < 0.05$ was considered statistically significant.

Supplementary Table S8. Correlations at baseline between weight, BMI, and nutrient intake and extraintestinal symptoms**.**

Baseline	**Weight**	**BMI**	**Energy**	**Carbohydrates**	**Protein**	**Fat**	**Fiber**	**Sucrose**	**Starch**
**Difficulties eating a meal**	Rs=(-0.106), p=0.092, q=0.131	Rs=(-0.048), p=0.448, q=0.552	Rs=(-0.077), p=0.222, q=0.444	Rs=(-0.038), p=0.549, q=0.915	Rs=(-0.061), p=0.334, q=0.477	Rs=(-0.067), p=0.284, q=0.568	Rs=(-0.082), p=0.195, q=0.325	Rs=0.049, p=0.435	Rs=(-0.071), p=0.261
**Headache**	Rs=0.094, p=0.135, q=0.169	Rs=0.175, p=0.005, q=0.012	Rs=(-0.029), p=0.646, q=0.807	Rs=0.004, p=0.948, q=0.971	Rs=(-0.050), p=0.431, q=0.539	Rs=(-0.021), p=0.734, q=0.918	Rs=(-0.120), p=0.055, q=0.183	Rs=0.040, p=0.520	Rs=0.009, p=0.881
**Back pain**	Rs=0.169, p=0.007, q=0.035	Rs=0.217, p<0.001, q=0.003	Rs=0.010, p=0.868, q=0.868	Rs=0.067, p=0.288, q=0.576	Rs=(-0.069), p=0.274, q=0.477	Rs=0.011, p=0.865, q=0.961	Rs=(-0.028), p=0.659, q=0.732	Rs=0.070, p=0.267	Rs=0.080, p=0.201
**Fatigue**	Rs=0.119, p=0.058, q=0.116	Rs=0.160, p=0.011, q=0.018	Rs=(-0.102), p=0.103, q=0.343	Rs=(-0.081), p=0.199, q=0.498	Rs=(-0.146), p=0.020, q=0.200	Rs=(-0.058), p=0.356, q=0.593	Rs=(-0.137), p=0.029, q=0.145	Rs=0.027, p=0.664	Rs=(-0.090), p=0.153
**Belching/excess wind**	Rs=(-0.034), p=0.253, q=0.281	Rs=0.038, p=0.552, q=0.552	Rs=0.144, p=0.021, q=0.210	Rs=0.130, p=0.039, q=0.39	Rs=0.064, p=0.311, q=0.477	Rs=0.123, p=0.050, q=0.387	Rs=0.158, p=0.012, q=0.120	Rs=0.048, p=0.444	Rs=0.050, p=0.429
**Reflux**	Rs=(-0.050), p=0.427, q=0.427	Rs=0.038, p=0.546, q=0.552	Rs=0.062, p=0.324, q=0.540	Rs=0.084, p=0.182, q=0.498	Rs=(-030), p=0.631, q=0.631	Rs=0.070, p=0.265, q=0.568	Rs=0.021, p=0.741, q=0.741	Rs=0.083, p=0.188	Rs=(-0.012), p=0.850
**Urinary urgency**	Rs=0.129, p=0.040, q=0.100	Rs=0.121, p=0.253, q=0.361	Rs=(-0.114), p=0.068, q=0.340	Rs=(-0.106), p=0.091, q=0.455	Rs=(-0.092), p=0.142, q=0.375	Rs=(-0.099), p=0.116, q=0.387	Rs=(-0.055), p=0.378, q=0.472	Rs=(-0.118), p=0.059	Rs=0.023, p=0.719
**Leg pain**	Rs=0.152, p=0.016, q=0.053	Rs=0.162, p=0.010, q=0.018	Rs=(-0.084), p=0.182 q=0.444	Rs=0.013, p=0.842, q=0.971	Rs=(-0.099), p=0.113, q=0.375	Rs=(-0.107, p=0.087, q=0.387	Rs=(-0.084), p=0.180, q=0.325	Rs=0.082, p=0.190	Rs=0.048, p=0.449
**Muscle/joint pain**	Rs=0.180, p=0.004, q=0.035	Rs=0.266, p<0.001, q=0.003	Rs=(-0.052), p=0.404, q=0.577	Rs=0.002, p=0.971, q=0.971	Rs=(-0.030), p=0.629, q=0.631	Rs=(-0.046, p=0.462, q=0.660	Rs=(-0.106), p=0.091, q=0.227	Rs=0.068, p=0.283	Rs=0.037, p=0.557
**Total extraintestinal IBS-SSS**	Rs=0.113, p=0.074, q=0.123	Rs=0.207, p<0.001, q=0.003	Rs=(-0.022), p=0.726, q=0.807	Rs=0.025, p=0.689, q=0.971	Rs(-0.091), p=0.150, q=0.375	Rs=0.000 ,p=0.999, q=0.999	Rs=(-0.059), p=0.351, q=0.472	Rs=0.069 ,p=0.275	Rs=0.031, p=0.622

BMI = body mass index. Specific and total extraintestinal symptoms were assessed by irritable bowel syndrome severity scoring system (IBS-SSS) [20]. There were as most 8 missing calculations. Spearman's correlation test. p-values were adjusted for false discovery rate (FDR) set at 5% according to the Benjamini─Hochberg method [33]. The FDR-adjusted q-values was the main result. q < 0.05 was considered statistically significant.

Supplementary Table S9. Correlations between differences (Δ) of weight, BMI, and nutrient intake and extraintestinal symptoms during the study.

Differences Δ	**Δ Weight**	**Δ BMI**	**Δ Energy**	**Δ Carbohydr**	**Δ Protein**	**Δ Fat**	**Δ Fiber**	**Δ Sucrose**	**Δ Starch**
**Difficulties eating a meal**	Rs=(-0.033) p=0.259, q=0.341	Rs=(-0.024) p=0.716, q=0.796	Rs=(-0.033), p=0.614, q=0.614	Rs=0.034, p=0.609, q=0.676	Rs=(-0.070), p=0.289	Rs=(-0.041), p=0.536, q=0.596	Rs=(-0.014), p=0.834 q=0.834	Rs=0.035, p=0.591 q=0.666	Rs=0.004, p=0.949
**Headache**	Rs=0.044, p=0.503, q=0.559	Rs=0.049, p=0.457, q=0.571	Rs=0.095, p=0.149, q=0.213	Rs=0.177, p=0.007, q=0.067	Rs=(-0.025), p=0.708	Rs=0.034, p=0.605, q=0.605	Rs=0.052, p=0.429 q=0.536	Rs=0.147, p=0.025 q=0.117	Rs=0.109, p=0.099
**Back pain**	Rs=0.110, p=0.094, q=0.235	Rs=0.119, p=0.088, q=0.196	Rs=0.227, p<0.001, q=0.010	Rs=0.158, p=0.016, q=0.067	Rs=0.100, p=0.129	Rs=0.195, p=0.003, q=0.030	Rs=0.038, p=0.562 q=0.624	Rs=0.095, p=0.232 q=0.387	Rs=0.114, p=0.082
**Fatigue**	Rs=0.086, p=0.190, q=0.317	Rs=0.093, p=0.156, q=0.260	Rs0.046, p=0.485, q=0.593	Rs=0.056, p=0.398, q=0.569	Rs=(-0.035), p=0.600	Rs=0.044, p=0.501, q=0.596	Rs=0.121, p=0.066, q=0.330	Rs=0.040, p=0.540 q=0.666	Rs=0.015, p=0.823
**Belching/excess wind**	Rs=0.133, p=0.043, q=0.197	Rs=0.142, p=0.031, q=0.127	Rs=0.180, p=0.006, q=0.030	Rs=0.153, p=0.020, q=0.067	Rs=0.046, p=0.488	Rs=0.094, p=0.153, q=0.382	Rs=0.142, p=0.030, q=0.300	Rs=0.035, p=0.599 q=0.666	Rs=0.098, p=0.136
**Reflux**	Rs=0.097, p=0.141, q=0.282	Rs=0.109, p=0.098, q=0.196	Rs=0.041, p=0.534, q=0.593	Rs=0.028, p=0.676, q=0.676	Rs=(-0.041), p=0.538	Rs=0.068, p=0.306, q=0.496	Rs=(-0.070), p=0.291 q=0.485	Rs=0.113, p=0.085 q=0.176	Rs=(-0.012), p=0.861
**Urinary urgency**	Rs=0.169, p=0.010, q=0.100	Rs=0.165, p=0.012, q=0.120	Rs=0.095, p=0.148, q=0.213	Rs=0.028, p=0.672, q=0.676	Rs=0.028, p=0.667	Rs=0.122, p=0.063, q=0.210	Rs=0.096, p=0.143 q=0.368	Rs=(-0.005), p=0.938 q=0.938	Rs=(-0.004), p=0.954
**Leg pain**	Rs=0.072, p=0.273, q=0.341	Rs=0.086, p=0.189, q=0.270	Rs=0.108, p=0.099, q=0.198	Rs=0.110, p=0.094, q=0.188	Rs=0.025, p=0.701	Rs=0.083, p=0.210, q=0.420	Rs=(-0.071), p=0.282 q=0.485	Rs=0.139, p=0.035 q=0.117	Rs=0.103, p=0.119
**Muscle/joint pain**	Rs=(-0.005) p=0.994, q=0.994	Rs=(-0.004), p=0.949, q=0.949	Rs=0.116, p=0.079, q=0.198	Rs=0.102, p=0.121, q=0.202	Rs=0.085, p=0.196	Rs=0.062, p=0.347, q=0.496	Rs=0.055, p=0.409 q=0.536	Rs=0.161, p=0.014 q=0.117	Rs=0.077, p=0.244
**Total extraintestinal IBS-SSS**	Rs=0.125, p=0.059, q=0.197	Rs=0.138, p=0.038, q=0.127	Rs=0.162, p=0.014, q=0.047	Rs=0.123, p=0.064, q=0.160	Rs=0.026, p=0.694	Rs=0.144, p=0.030, q=0.150	Rs=0.096, p=0.147 q=0.368	Rs=0.113, p=0.088 q=0.176	Rs=0.066, p=0.320

BMI = body mass index. Specific and total extraintestinal symptoms were assessed by irritable bowel syndrome-severity scoring system (IBS-SSS) [20]. There were 36 missing calculations. Spearman's correlation test. p-values were adjusted for false discovery rate (FDR) set at 5% according to the Benjamini─Hochberg method [33]. The FDR-adjusted q-values was the main result. q < 0.05 was considered statistically significant.

Supplementary Table S10. Correlations between weight/BMI, nutrients, and lipid values**.**

	**Cholesterol**	**LDL cholesterol**	**HDL cholesterol**	**Non-HDL cholesterol**
Baseline				
**Weight** (kg)	Rs=0.064, p=0.314, q=0.314	Rs=0.153, p=0.015, q=0.020	Rs=(-0.423), p<0.001, q=0.002	Rs=0.229, p<0.001, q=0.002
**BMI** (kg/m^2)	Rs=0.142, p=0.025, q=0.025	Rs=0.228, p<0.001, q=0.001	Rs=(-.408), p<0.001, q=0.001	Rs=0.309, p<0.001, q=0.001
**Energy** (kcal)	Rs=0.034, p=0.596	Rs=0.007, p=0.918	Rs=(-0.003), p=0.961	Rs=0.041, p=0.523
**Carbohydrates** (g)	Rs=(-0.038), p=0.548	Rs=(-0.084), p=0.185	Rs=(-0.110), p=0.081	Rs=(-0.001), p=0.988
**Protein** (g)	Rs=(-0.053), p=0.404	Rs=(-0.084), p=0.183	Rs=(-0.041), p=0.520	Rs=(-0.054), p=0.402
**Fat** (g)	Rs=0.078, p=0.216	Rs=0.079, p=0.210	Rs=0.40, p=0.524	Rs=0.078, p=0.227
**Fiber** (g)	Rs=(-0.026), p=0.686	Rs=(-0.052), p=0.413	Rs=(-0.026), p=0.679	Rs=(-0.007), p=0.917
**Sucrose** (g)	Rs=(-0.033), p=0.604	Rs=(-0.052), p=0.412	Rs=0.050, p=0.429	Rs=(-0.059), p=0.362
**Starch** (g)	Rs=(-0.088), p=0.165, q=0.220	Rs=(-0.224), p<0.001, q=0.004	Rs=(-0.186), p=0.003, q=0.006	Rs=(-0.016), p=0.806, q=0.806
Differences (Δ)	**ΔCholesterol**	**ΔLDL cholesterol**	**ΔHDL cholesterol**	**ΔNon-HDL cholesterol**
**Weight** (kg)	Rs=0.089, p=0.184, q=0.368	Rs=(-0.035), p=0.601, q=0.747	Rs=0.182, p=0.006, q=0.024	Rs=0.022, p=0.747, q=0.747
**BMI** (kg/m^2)	Rs=0.082, p=0.218, q=0.436	Rs=(-0.039), p=0.558, q=0.744	Rs=0.180, p=0.007, q=0.028	Rs=0.013, p=0.850, q=0.850
**Energy** (kcal)	Rs=0.103, p=0.126	Rs=0.066, p=0.322	Rs=0.101, p=0.132	Rs=0.071, p=0.298
**Carbohydrates** (g)	Rs=0.019, p=0.777	Rs=(-0.029), p=0.670	Rs=0.040, p=0.550	Rs=0.010, p=0.880
**Protein** (g)	Rs=0.017, p=0.806	Rs=0.008, p=0.909	Rs=0.016, p=0.808	Rs=(-0.004), p=0.949
**Fat** (g)	Rs=0.087, p=0.148	Ra=0.080, p=0.230	Rs=0.104, p=0.121	Rs=0.058, p=0.393
**Fiber** (g)	Rs=0.110, p=0.100	Rs=0.110, p=0.102	Rs=0.071, p=0.293	Rs=0.091, p=0.180
**Sucrose** (g)	Rs=(-0.012), p=0.859	Rs=(-0.003), p=0.969	Rs=(-0.021), p=0.753	Rs=(-0.003), p=0.968
**Starch** (g)	Rs=0.012, p=0.859	Rs=(-0.034), p=0.615	Rs=0.027, p=0.688	Rs=(-0.001), p=0.994

BMI = body mass index; HDL = high-density lipoprotein; LDL = low-density lipoprotein. There were 10 missing calculations at baseline and 35 in the difference calculations. Spearman's correlation test. p-values were adjusted for false discovery rate (FDR) set at 5% according to the Benjamini–Hochberg method [33]. The FDR-adjusted q-values was the main result. q < 0.05 was considered statistically significant.

Supplementary Table S11. Significant associations between weight and nutrient intake, and lipid levels and symptoms at baseline.

Dependent variables	**Weight**	**Carbohydrates**	**Protein**	**Fat**	**Fiber**
**Cholesterol**				β:0.006;95%CI:0.000-0.011;p=0.033	
**LDL**	β:0.012;95%CI:0.003-0.020;p=0.008	β:-0.003;95%CI:-0.005-0.000;p=0.038	β:-0.007;95%CI:-0.012-(-0.001);p=0.028	β:0.008;95%CI:0.003-0.013;p=0.002	
**HDL**	β:-0.01;95% CI:-0.013-(-0.007);p<0.001				
**Non-HDL**	β:0.017;95%CI:0.009-0.026;p<0.001				
**Constipation**	β:-0.549;95%CI:-0.824-(-0.273);p<0.001				
**Intestinal symptoms´ influence on daily life**	β:-0.190; 95%CI:-0.374-(-0.007);p=0.042				
**Psychological well-being**		β:0.084;95%CI:0.015-0.153;p=0.018			β:-0.601;95%CI:-1.103-(-0.098);p=0.019
**Headache**	β:-0.683;95%CI:-1.232-(-0.133);p=0.015				
**Back pain**	β:0.0342;95%CI:0.072-0.613;p=0.013		β:-0.200;95%CI:-0.395-(-0.006);p=0.044		
**Fatigue**	β:0.254;95%CI:0.021-0.487;p=0.033				
**Urinary urgency**	β:0.351;95%CI:0.078-0.624;p=0.012	β:-0.091;95%CI:-0.176-(-0.006);p=0.037			
**Leg pain**	β:0.340;95%CI:0.136-0.544;p=0.001		β:-0.175;95%CI:-0.322-(-0.029);p=0.019		
**Muscle/joint pain**	β:0.361;95%CI:0.080-0.642;p=0.012				

HDL = high-density lipoprotein; LDL = low-density lipoprotein. Specific and total gastrointestinal and extraintestinal symptoms were assessed by the irritable bowel syndrome severity scoring system (IBS-SSS) and visual analog scale for irritable bowel syndrome (VAS-IBS) [20, 30]. There were 8 missing calculations for symptoms and 12 for lipid values. Generalized linear model was used for calculations of associations between lipids and symptoms (dependent variables) and nutrients in a model with weight, carbohydrates, protein, fat, and fiber as independent variables with values given as β-value and 95% confidence interval (CI). p<0.05 was considered statistically significant.

Supplementary Table S12. Significant associations between differences (Δ) in weight and nutrient intake, and lipid levels and symptoms.

HDL = high-density lipoprotein. Specific and total gastrointestinal and extraintestinal symptoms were assessed by the irritable bowel syndrome-

Dependent variables	**ΔWeight**	**ΔCarbohydrates**	**ΔProtein**	**ΔFat**	**ΔFiber**
**ΔHDL cholesterol**	β:0.017;95% CI:0.006-0.029;p=0.004				
**ΔConstipation**			β:0.231;95% CI:0.069-0.393;p=0.005	β:-1.116;95% CI:-0.219-(-0.012);p=0.028	
**ΔIntestinal symptoms´ influence on daily life**	β:2.022;95% CI:0.015-4.028;p=0.048		β:-.164;95% CI:-0.326-(-0.001);p=0.048		
**ΔHeadache**		β:0.062;95% CI:0.018-0.106;p=0.006			
**ΔFatigue**	β:2.576;95% CI:0.853-4.299;p=0.003				
**ΔReflux**	β:1.836;95% CI:0.099-3.573;p=0.038				
**ΔUrinary urgency**	β:2.514;95%CI:0.739-4.290;p=0.006				
**ΔLeg pain**				β:0.067;95% CI:0.002-0.133;p=0.045	β:-0.359;95% CI:-0.718-0.000;p=0.050
**ΔTotal IBS-SSS**	β:7.241;95% CI:0.071-14.412;p=0.048	β:0.218;95% CI:0.017-0.420;p=0.034			
**ΔTotal extraintestinal IBS-SSS**	β:5.169;95% CI:0.807-9.530;p=0.020				

severity scoring system (IBS-SSS) and visual analog scale for irritable bowel syndrome (VAS-IBS) [20, 30]. There were 36 missing calculations for symptoms and 43 for lipid levels. Generalized linear model was used for calculations of associations between lipids and symptoms (dependent variables) and nutrients in a model with weight, carbohydrates, protein, fat, and fiber as independent variables with values given as β-value and 95% confidence interval (CI). p<0.05 was considered statistically significant.

Supplementary Table S13. Significant associations between BMI and nutrient intake, and lipid levels and symptoms at baseline.

Dependent variables	**BMI**	**Carbohydrates**	**Protein**	**Fat**	**Fiber**
**Cholesterol**	β:0.029;95%CI:0.003-0.056;p=0.028				
**LDL cholesterol**	β:0.047;95%CI:0.021-0.073;p<0.001	β:-0.003;95%CI:-0.005-0.000;p=0.027		β:0.008;95%CI:0.003-0.013;p=0.002	
**HDL cholesterol**	β:-0.027;95%CI:-0.036-(-0.018);p<0.001				
**Non-HDL cholesterol**	β:0.061;95%CI:0.035-0.088;p<0.001				
**Bloating**	β:-0.142;95%CI:-0.279-(-0.004);p=0.043				
**Psychological well-being**		β:0.083;95%CI:0.014-0.153;p=0.018			β:-0.587;95%CI:-1.088-(-0.086);p=0.022
**Headache**	β:0.928;95%CI:0.199-1.658;p=0.013				β:-0.630;95%CI:-1.171-(-0.089);p=0.022
**Back pain**	β:1.117;95%CI:-0.831-396;p=0.008				
**Fatigue**	β:1.022;95%CI:0.313-1.731;p=0.005				
**Urinary urgency**	β:0.961;95%CI:0.124-1.799;p=0.024	β:-0.091;95%CI:-0.177-(-0.006);p=0.037			
**Leg pain**	β:1.032;95%CI:0.408-1.656;p=0.001		β:-0.146;95%CI:-0.292-(8.021E-5);p=0.050		
**Muscle/joint pain**	β:1.656;95%CI:0.809-2.503;p<0.001				
**Total extraintestinal IBS-SSS**	β:3.213;95%CI:1.230-5.196;p=0.001				

HDL = high-density lipoprotein; LDL = low-density lipoprotein. Specific and total gastrointestinal and extraintestinal symptoms were assessed by the irritable bowel syndrome severity scoring system (IBS-SSS) and visual analog scale for irritable bowel syndrome (VAS-IBS) [20, 30]. There were 8 missing calculations for symptoms and 12 for lipid values. Generalized linear model was used for calculations of associations between lipids and symptoms (dependent variables) and nutrients in a model with body mass index (BMI), carbohydrates, protein, fat, and fiber as independent variables with values given as β-value and 95% confidence interval (CI) for. p<0.05 was considered statistically significant.

Supplementary Table S14. Significant associations between differences (Δ) in BMI and nutrient intake, and lipid levels and symptoms.

Dependent variables	ΔBMI	ΔCarbohydrates	ΔProtein	ΔFat	ΔFiber
**HDL cholesterol**	β:0.046;95%CI:0.013-0.079;p=0.006				
**Constipation**			β:0.227;95%CI:0.065-0.389;p=0.006	β:-0.115;95%CI:0.218-(-0.012);p=0.029	
**Intestinal symptoms´ influence on daily life**	β:6.447;95%CI:0.785-12.109;p=0.026		β:-0.168;95%CI:-0.331-(-0.006);p=0.042		
**Headache**		β:0.06195%CI:0.017-0.105;p=0.006			
**Fatigue**	β:8.036;95%CI:3.182-12.890;p=0.001				β:0.735;95%CI:0.005-1.466;p=0.048
**Reflux**	β:5.226;95%CI:0.312-10.140;p=0.037				
**Urinary urgency**	β:6.949;95%CI:1.922-11.975;p=0.007				
**Leg pain**				β:0.067;95%CI:0.002-0.133;p=0.045	β:-0.363;95%CI:-0.722-(-0.004;p=0.048
**Total IBS-SSS**	β:22.691;95%CI:2.447-42.934;p=0.028	β:0.218;95%CI:0.018-0.419;p=0.033			
**Total extraintestinal IBS-SSS**	β:16.193;95%CI:3.889-28.497;p=0.010				

HDL = high-density lipoprotein. Specific and total gastrointestinal and extraintestinal symptoms were assessed by the irritable bowel syndrome-severity scoring system (IBS-SSS) and visual analog scale for irritable bowel syndrome (VAS-IBS) [20, 30]. There were 36 missing calculations for symptoms and 43 for lipid levels. Generalized linear model was used for calculations of associations between lipids and symptoms (dependent variables) and nutrients in a model with body mass index (BMI), carbohydrates, protein, fat, and fiber as independent variables with values given as β-value and 95% confidence interval (CI). $p<0.05$ was considered statistically significant.

Supplementary Table S15. Significant associations between BMI, sucrose, and starch, and lipid levels and symptoms at baseline.

Dependent variables	**BMI**	**Sucrose**	**Starch**
**Cholesterol**	β: 0.031; 95%: 0.005-0.058; p=0.019		
**LDL cholesterol**	β: 0.047; 95% CI: 0.022-0.073; p<0.001		β: -0.006; 95%CI: -0.009-(-0.002); p=0.001
**HDL cholesterol**	β: -0.029; 95%CI: -0.037-(-0.020); p<0.001		β: -0.002; 95% CI: -0.003-(-0.001); p=0.004
**Non-HDL cholesterol**	β: 0.065; 95% CI: 0.039-0.091; p<0.001		
**Constipation**	β: -0.965; 95%CI: -1.815-(-0.114); p=0.026		
**Headache**	β: 1.034; 95% CI: 0.303-1.766; p=0.006		
**Back pain**	β: 1.199; 95% CI: 0.376-2.023; p=0.004		
**Fatigue**	β: 0.987; 95%CI: 0.278-1.695); p=0.006		
**Belching/excess wind**			
**Urinary urgency**	β: 0.919; 95%CI: 0.090-1.749; p=0.030	β: -0.258; 95%CI: -0.446-(-069); p=0.007	
**Leg pain**	β: 1.017; 95%CI: 0.391-1.642; p=0.001		
**Muscle/joint pain**	β: 1.679; 95%CI: 0.835-2.522; p<0.001		
**Total extraintestinal IBS-SSS**	β: 3.262; 95%CI: 1.281-5.243; p=0.001		

HDL = high-density lipoprotein; LDL = low-density lipoprotein. Specific and total gastrointestinal and extraintestinal symptoms were assessed by irritable bowel syndrome severity scoring system (IBS-SSS) and visual analog scale for irritable bowel syndrome (VAS-IBS) [20, 30]. There were 8 missing calculations for symptoms and 12 for lipid values. Generalized linear model was used for calculations of associations between lipids and symptoms (dependent variables) and nutrients in a model with body mass index (BMI), sucrose, and starch as independent variables with values given as β-value and 95% confidence interval (CI). $p<0.05$ was considered statistically significant.

Supplementary Table S16. Significant associations between differences (∆) in body mass index (BMI), sucrose, and starch, and lipid levels and symptoms.

Dependent variables	**∆BMI**	**∆Sucrose**	**∆Starch**
**HDL cholesterol**	β: 0.044; 95% CI: 0.011-0.077); p=0.010		
**Intestinal symptoms´ influence on daily life**	β: 5.979; 95% CI: 0.208-11.751); p=0.042		
**Fatigue**	β: 7.188; 95%CI: 2.255-12.122; p=0.004		
**Reflux**	β: 5.089; 95%CI: 0.120-10.057; p=0.045		
**Urinary urgency**	β: 6.170; 95%CI: 1.115-11.224; p=0.017		
**Total IBS-SSS**	β: 22.998; 95%CI: 2.300-43.695; p=0.029	β: 0.589; 95%CI: 0.027-1.151; p=0.040	
**Total extraintestinal IBS-SSS**	β: 16.298; 95%CI: 3.899-28.697; p=0.010		

HDL = high-density lipoprotein. Specific and total gastrointestinal and extraintestinal symptoms were assessed by irritable bowel syndrome severity scoring system (IBS-SSS) and visual analog scale for irritable bowel syndrome (VAS-IBS) [20, 30]. There were 36 missing calculations for symptoms and 43 for lipid values. Generalized linear model was used for calculations of associations between lipids and symptoms (dependent variables) and nutrients in a model with BMI, sucrose, and starch as independent variables with values given as β-value and 95% confidence interval (CI). $p<0.05$ was considered statistically significant.
